# Bio-based resources: systemic & circular solutions for (agro)environmental services

**DOI:** 10.1039/d4ra03506b

**Published:** 2024-07-25

**Authors:** Gabrijel Ondrasek, Cristian Meriño-Gergichevich, Carlos Manterola-Barroso, Alex Seguel Fuentealba, Sebastián Meier Romero, Radovan Savić, Sarvamangala S. Cholin, Jelena Horvatinec

**Affiliations:** a University of Zagreb, Faculty of Agriculture Svetosimunska c. 25 Republic of Croatia gondrasek@agr.hr; b Scientific and Technological Bioresources Nucleus (BIOREN-UFRO), Universidad de La Frontera Temuco Chile cristian.merino@ufrontera.cl; c Laboratory of Physiology and Plant Nutrition for Fruit Trees, Faculty of Agricultural Sciences and Environment, Universidad de La Frontera Temuco Chile; d Laboratory of Soil Fertility, Faculty of Agricultural Sciences and Environment, Universidad de La Frontera Temuco Chile; e Department of Agricultural Production, Faculty of Agricultural Sciences and Environment, Universidad de La Frontera Temuco Chile; f Doctoral Program in Science of Natural Resources, Universidad de La Frontera Temuco Chile carlosignacio.manterola@ufrontera.cl; g Departamento de Ciencias Agronómicas y Recursos Naturales, Facultad de Ciencias Agropecuarias y Medioambiente, Universidad de La Frontera Temuco Chile alex.seguel@ufrontera.cl; h Instituto de Investigaciones Agropecuarias, INIA Carillanca Temuco Chile sebastian.meier@inia.cl; i Faculty of Agriculture, University of Novi Sad Trg D. Obradovica 1 21000 Novi Sad Serbia radovan.savic@polj.uns.ac.rs; j Plant Molecular Biology Lab (DBT-BIOCARe), Department of Biotechnology & Crop Improvement, College of Horticulture, University of Horticultural Sciences Bagalkot 587103 Karnataka India sarucholin@gmail.com; k University of Horticultural Sciences Bagalkot 587103 Karnataka India; l School of Agronomy, Faculty of Sciences, Engineering, and Technology, Universidad Mayor Temuco Chile

## Abstract

The global promotion of decarbonisation through the circular solutions and (re)use of bio-based resources (BBR), *i.e.* waste streams, notably from the agricultural, forest and municipal sectors has steadily increased in recent decades. Among the transformative solutions offered by BBR, biosolids (BS), biochars (BC), and bioashes (BA) specifically attract scientific attention due to their highly complex organo-mineral matrices, which present significant potential for recovery in the agro-/forest-ecosystems. These materials enhance various soil (i) chemical (pH, macro/micro nutrient concentrations, organic matter content), (ii) physical (porosity, water–air relations, compaction) or (iii) microbial (diversity, activity) properties. Furthermore, some of transformed BBR contribute to a multitude of environmental services such as the remediation of contaminated sites and wastewater treatment, employing cost-effective and eco-friendly approaches that align with circular economy/waste management principles, ultimately contributing to climate change mitigation. However, several challenges impede the widespread utilization/transformation of BBR, including technological limitations in processing and application, concerns about contamination (*e.g.*, PAHs, PCBs, micro/nano plastics present in BS), toxicity issues (*e.g.*, heavy metals in BA or nanoparticles in BC), and regulatory constraints (*e.g.*, non-uniform regulations governing the reuse of BA and BS). Addressing these challenges demands an interdisciplinary and intersectoral approach to fully unlock the potential of BBR in sustainable decarbonisation efforts.

## Abstract 2

Transformative solutions for key bio-based resources: environmental functions, implications & mechanisms of action.

## Graphical abstract

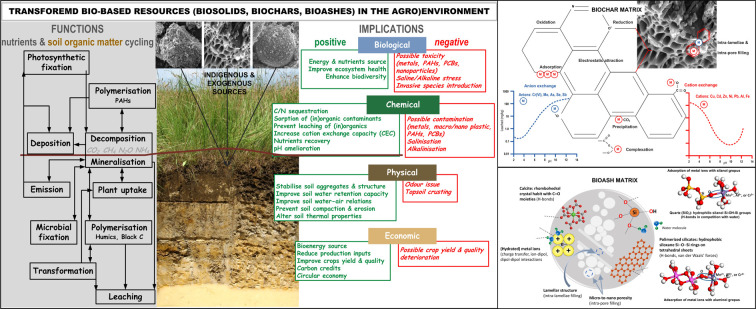

## 1. Introduction

**Fig. 1:**
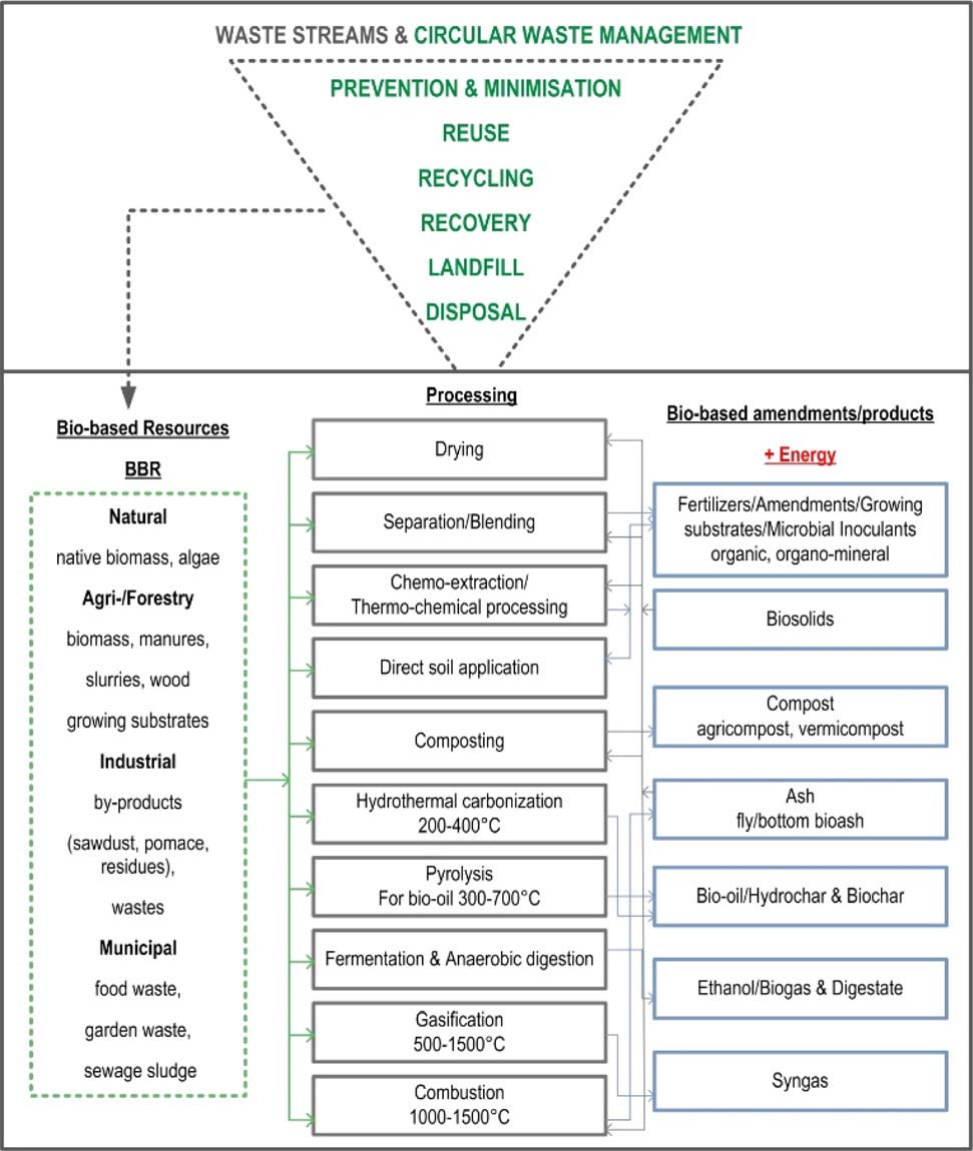
Principles of circular waste management and transforming of bio-based resources (BBR) into the value-added products.^5–7^

Waste management has become a pressing global concern due to population growth and urbanization^1^ and the negative impacts on the environmental quality and public health.^2^ The modern consumer-driven economy has led to an increase in waste generation; the constant production of disposable goods, packaging, and electronics is adding to the waste crisis.^3^ The environmental challenges associated with waste management are profound, given their adverse effects on air and water quality, soil health, and the greenhouse gas (GHG) emission.^4^ Thus, the need for effective and more sustainable, *i.e.*, circular waste management solutions (Fig. 1) has become imperative. Namely, contemporary waste streams contain valuable resources (metals, organics, fibres, minerals) that are being lost to landfills instead of being recycled or reused (Fig. 1). Consequently, contemporary environmental policies and strategies in their long-term vision have incorporated numerous goals to transform the agro-industrial sectors into low-environmental impact, climate-neutral, resource-efficient, and circular solutions by transforming and utilizing different waste materials, *i.e.*, resources (Fig. 1). Among different such waste streams, bio-based resources (BBR) emerges as a prominent concern due to their intricate composition,^8^ diverse sources, and substantial environmental implications,^9^ necessitating tailored management approaches and innovative utilization strategies (Fig. 1).

BBR encompass a broad spectrum of organic materials derived from native sources, including agricultural and forestry by-products/residues, organic residues from food processing, municipal waste and other bio-based materials (Fig. 1). Recognizing the potential of BBR as a valuable pool for various applications, efforts are being made to develop innovative technologies and strategies to efficiently manage and utilize them more sustainably.^10^ By harnessing the potential of BBR it is possible to not only mitigate environmental impacts (*e.g.*, GHG emission), but also create new economic opportunities and contribute to the transition towards a more sustainable and circular (bio)economy (Fig. 1).

The European Union (EU) has established several obligations with regards to waste management and energy targets, as outlined in the EU Waste Framework Directive,^11^ Renewable Energy Directive,^12^ European Green Deal,^13^ Farm to Fork strategy,^13^ and the Circular Economy Action Plan.^13^ Some of the key obligations for EU member states include: (i) reduction of waste generation, (ii) separation/reusing of waste, (iii) landfill targets (*e.g.* states are required to reduce the amount of biodegradable municipal waste sent to landfill by up to 35% by 2020, and to reduce landfilling to a maximum of 10% of municipal waste by 2030 (ref. 14, iv) waste treatment standards (*e.g.* incinerators and landfill sites must meet strict environmental and operational standards to minimize the impact on human health and the environment), (v) promotion of renewables (*e.g.* biomass plant facilities), and (vi) reach at least a 27% share of renewable energy by 2030. Following on these targets, utilization of biomass has increased significantly in recent decades.^15^ The promotion of the benefits and opportunities of renewables has led to a significant increase in the number of biomass plant facilities, consequently rising the generation of valuable BBR, particularly bioash (BA) by-product^16^ (Fig. 1). Currently, 70% of BA is landfilled, 20% is used as a soil conditioner in agri-/forest sector, and 10% is used for miscellaneous applications.^5^ The significant cost of BA management (100–500 EUR per t) with the landfill costs expected to increase in the future as a consequence of waste tax or disposal fee, difficulties in acquiring new landfill sites, and the stricter EU landfill directives.^17^ Thus, it is essential to identify environmentally sustainable and economically viable approaches for applying or transforming BA to value-added solutions.

The situation with other important BBR stream, such as municipal solid waste (MSW), becomes even more complex. MSW is dominated by several waste types (food waste, yard waste, paper/cardboard and plastics),^18^ and its qualitative–quantity properties and transformative routes vary greatly among: (i) countries (developing *vs.* developed) and (ii) regions (*e.g.*, mostly naturally-based waste materials in the rural areas *vs.* predominantly artificially-based waste in urban/industrial regions). For instance, in low and lower-middle income countries, organic waste typically comprises 53–56% of MSW, with annual yield of 220–290 kg per capita.^19^ However, exceptions like Sri Lanka are notable, where food waste represents 50–76% of the total MSW.^20^ Food waste triggers substantial economic, environmental, and social implications, necessitating the urgent implementation of reduction strategies.^21^ It is estimated that ∼88 Mt of food waste is generated in EU each year.^14,22,23^ Reducing food waste in the EU by 30% could result in annual savings of €120 billion, creating jobs and improving resource-use efficiency.^14,22,23^ To address the economic impact of food waste in EU countries, the target is to reduce food waste by 50% by 2030, as a part of the EU's broader efforts to a circular (bio)economy transition.^13^ Conversely, in developed countries MSW is dominated by other waste materials (*e.g.*, plastic, paper) with annual yields reaching up to 420 and even 780 kg per capita in upper-middle income and high-income countries, respectively.^19^ According to the most recent statistical consolidated report, in 2022, the EU produced 513 kg of MSW per capita, with 49% of that waste being recycled through material recycling (30%) and composting (18%), followed by incineration (26%), landfilling (23%) and other treatments (3%) (Table 1). According to the same source in the EU there has been a significant change in municipal waste treatments in last several decades. For instance, in the 1995–2022 period incineration has increased by 98%, from 30 Mt (70 kg per capita) to 59 Mt (133 kg per capita), mostly at the expanse on reduced landfill, which dropped by 56%, from 121 Mt (286 kg per capita) to 53 Mt (118 kg per capita) (Table 1).

**Table 1:** Municipal solid waste (MSW) management in the EU, 1995–2022 (ref. 19)

Management	1995		2000		2005		2010		2015		2020		2022		Change 2022/1995 (%)	
	Mt	kg per cap.	Mt	kg per cap.	Mt	kg per cap.	Mt	kg per cap.	Mt	kg per cap.	Mt	kg per cap.	Mt	kg per cap.	Mt	kg per cap.
Landfill	121	286	112	262	88	202	79	178	57	127	54	121	53	118	−56	−59
Incineration	30	70	36	84	45	103	53	121	57	128	62	138	59	133	98	91
Recycling	23	54	38	87	46	105	55	125	63	141	69	154	68	153	196	181
Composting	14	33	23	53	26	59	29	66	33	75	43	96	43	96	203	187
Other	10	23	11	27	16	37	6	13	4	9	4	9	6	14	−37	−40
Total	198	467	220	513	220	506	222	503	213	480	232	519	229	513		

It is proposed that the bulk of non-recyclable MSW is optimally thermally processed by pyrolysis or incineration, primarily resulting in: (i) energy (thermal, electrical), (ii) fuel, and (iii) high-C (biochar) or (iv) low-C mineral residual (ash) matrix^24^ (Fig. 1). The conversion of BBR into biochar (BC) provides significant environmental benefits, including enhanced soil fertility/water–air relations, C sequestration, higher crop yields and mitigation of climate change impacts (more in Section 4).

At the global scale, 216 Mt of annually collected MSW undergo incineration in approximately 2000 thermal plant facilities mostly in high-income (Japan, EU, China, USA) countries.^19^ In the EU utilization of MSW-derived ash is already practiced, but to a small extent (*e.g.*, in Belgium, Netherlands, Luxemburg, France, Germany) and mainly for low-value applications (*e.g.*, road sub-base material).^25^ As avoiding of landfilling completely is still out of reach, it represents significant environmental and human health risk due to a wide range of organo-mineral contaminants in the MSW-derived ash matrix.^11^ In particular, incineration is commonly applied for sewage sludge management, often reffered to as biosolids (BS), which originate from the wastewater treatment.^26^ BS management poses a persistent challenge as comprehensive solutions for this complex matrix and proper regulations are still lacking (more in Section 2). Most of the world's BS-derived ash is still landfilled at a significant additional cost to the utilities given on its classification as a potential hazardous waste (more in Section 2).

In recent decades BBR from the municipal, agricultural and forest sectors has steadily increased, and among the transformative solutions offered by them, BS, BC, and BA have attracted increasing interest as they are recognized to be valuable sources of macro and micronutrients (N, P, K, Ca, Mg, Zn, Cu, Mo, Mn), minerals (silica, struvite, limestone, dolomite), and organics (amino acids, proteins, humates, fulvates).^27,28^ The latest regulations on waste and wastewater management^29^ promote the utilization of reclaimed P, N, K and trace elements in fertilizers, necessitating their large-scale implementation. One effective approach to minimize resource consumption and emissions involves the utilization and transformation of BBR into value-added products, thereby reducing reliance on synthetic fertilizers, transportation, and energy, while also generating added economic value.^30^ Thus, it is believed that next-generation products (*e.g.*, soil conditioners, fertilizers, bio-composites) and technological processes (*e.g.*, slow/catalytic pyrolysis, nano-coating) will utilize BBR as multi-source waste streams, aiming to have ecological (green) and economical (smart) impacts on recycling, reusing, and repurposing waste streams to achieve cost-effective and circular waste management, reducing landfilling (Fig. 1), as critically discussed in this review.

## 2. Biosolids (BS): resources of nutrients & organics

**Fig. 2:**
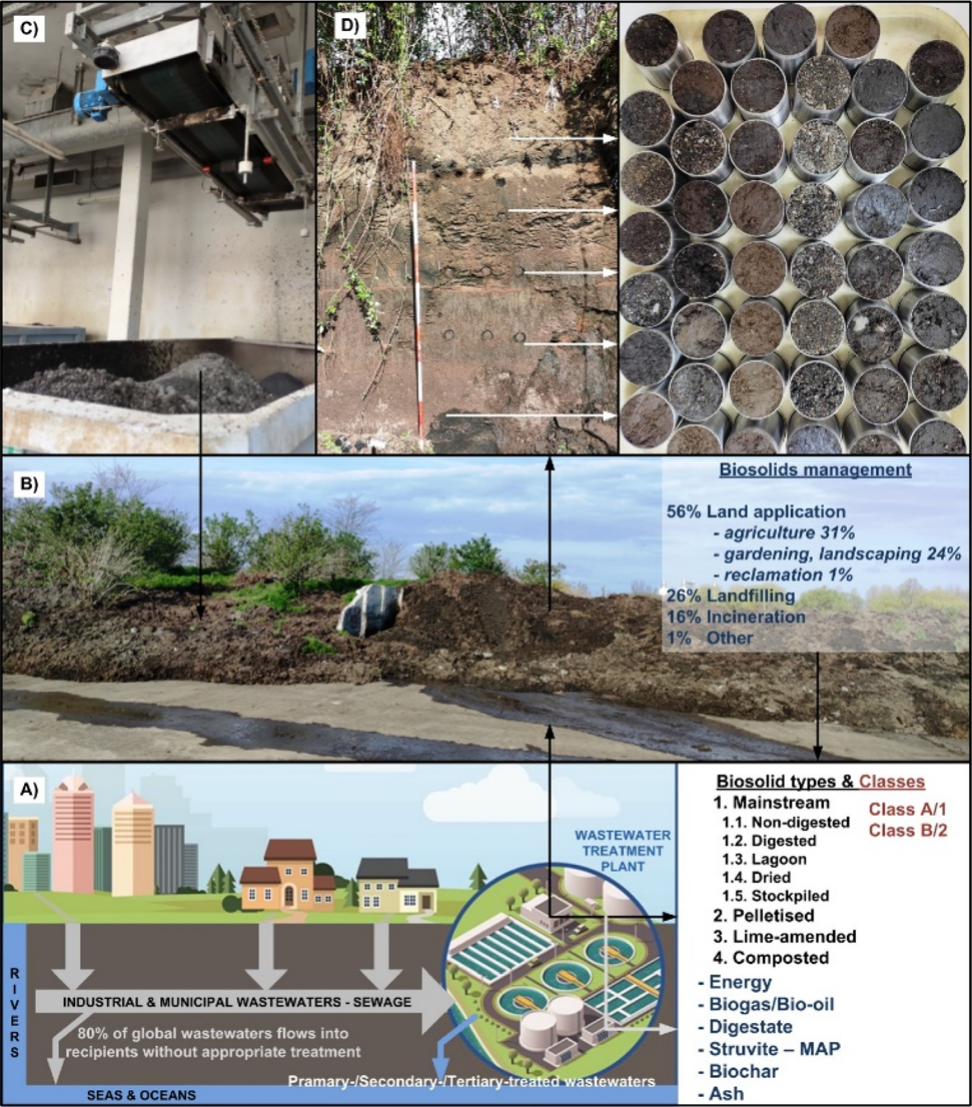
Schematic presentation of a wastewater treatment plant as a resource of biosolids (BS) and value-added (co)products (A). Sewage sludge landfill at Zagreb Wastewater Ltd (B). Liming of fresh sewage sludge (C). Sampling of aged sewage sludge in undisturbed conditions (D). Reproduced (adapted) with permission from ref. 32. Copyright© 2023, Uni. of Zagreb, ref. 33 Copyright© 2023, Springer Nature.

BS represent a complex organo-mineral by-product of the wastewater (sewage) treatment plant facilities,^31^ generated from the separation of solid sewage sludge from liquid wastewater, undergoing mechanical (physical), biological and/or chemical treatments^31^ (Fig. 1) to reduce pathogens, diminish vector attraction and stabilize organic matter (Fig. 2). Recent studies indicate that ∼80% of global wastewater is released into the environment without appropriate treatment,^6^ resulting in the current global production of 100–125 Mt of wet BS, which is projected to increase to 150–200 Mt by 2025.^34^

BS are generally characterized by their high content of solids (15–90%), depending on the age, profile position (Fig. 2), composed dominantly of organic matter (OM) and macro/micronutrients (P, N, K, Zn, Fe, Cu, B, Mo, Mn),^7^ making them suitable resources for amelioration of organically-depleted and nutrient-deficient land resources in agroecosystems (Fig. 2). Fertilizers for conventional agri-food production primarily contain essential nutrients (N, P, K, Ca, Mg and S) in different natural or synthetic forms, such as anhydrous ammonia (NH_3_), urea (CH_4_N_2_O), urea-ammonium nitrate, Ca-/Mg-ammonium nitrate, rock phosphate, mono-/di-ammonium phosphate, superphosphate, Ca phosphate hydroxyapatite, struvite, potash (KCl), potassium sulphate, *etc.*^35^ In 2020, a total of 147 Mt of NH_3_, 219 Mt of phosphate, and 44 Mt of KCl were synthetically produced or mined.^36^ However, recent estimates suggest that the municipal wastewater generated worldwide carries sufficient nutrients to substitute for 25% of N and 15% of P currently applied as synthetic fertilizers on agroecosystems,^37^ representing such waste stream a valuable source of nutrients (Fig. 2). For instance, in the urine and slurry under increased pH reaction and concentration of main ions is facilitated the precipitation of a mineral struvite (magnesium ammonium phosphate hexahydrate; MgNH_4_PO_4_·6H_2_O),^38^ known as MAP, which can be recovered from the wastewater streams as a complex fertiliser.^39^ In addition, different approaches have been applied to recover potassium struvite (MgKPO_4_·6H_2_O) from other waste streams, including a pig slurry following (de)nitrification and extraction from crop residues.^36^ For instance, struvite precipitation from wastewater has the capability to produce on a daily basis 17.3 kg of struvite from 1000 m^3^ of sewage.^40^ Hence, struvite mining from municipal effluents/biosolids offers numerous benefits, including prevention of hydro-resources from nutrient pollution (eutrophication), reducing the need for synthetic fertilizers and GHG emission, improving soil fertility, and promoting sustainable and circular waste management integrated with agriculture practices. For example, synthetic N fertilizers, generated through the energy-intensive Haber-Bosch process (reduction of atmospheric N with H to NH_3_), account for 1.8% of global energy consumption and contribute 1.8% to global CO_2_ emissions.^36^ Additionally, the recovery of struvite helps to conserve valuable phosphate rocks as a finite non-renewable resource,^41^ contributing to the circular bioeconomy by harnessing bio-based waste into a valuable resource for agri-food production.

### 2.1 Biosolids (BS) & restriction in use

Additionally, BS have the capacity to enhance physical pedovariables, improving water–air relations, reducing the bulk density and increasing soil porosity,^42^ particularly in heavy-textured (clayey) and poorly structured soils. However, BS have physically, chemically and biologically very complex and divers matrices,^43^ making them challenging for effective use as standardised soil amendments. For instance, a summary statistic of nearly 500 samples of aged BS (from one to over 10 years) taken in undisturbed conditions by inox cores (100 dm^3^) revealed significant heterogeneity (*p* < 0.001) in their physical properties. The average mass of fresh BS ranged from 103.21 g/100 cm^3^ (surface layer at a height of 200 cm) to 200.84 g/100 cm^3^ (layer at a height of 40 cm), whereas dry samples (at 105 °C) exhibited even greater variability, ranging from 48.36 g/100 cm^3^ (at a height of 40 cm) to 162.95 g/100 cm^3^ (at a height of 120 cm)^32^ (Fig. 2D).

According to the recent report, out of ∼3.8 M dry metric tons of BS generated in the USA, the majority (56%) has been applied to land areas (31% in agriculture and 25% in home gardens, landscapes, forestry), following landfilling (27%), incineration (16%) and other treatments^33^ (Fig. 2B). Similarly, the European Commission's report indicates that nearly 40% of BS produced in the European Union is recycled in the agri-sector.^44^ In Australia, out of the 350 000 t dry BS generated annually even 83% is beneficially utilized, primarily within agroecosystems (75%), followed by stockpiling (13%), while the rest is allocated to land reclamation, landscaping, landfilling, and other purposes.^45^ However, prior to land application, sewage sludge, *i.e.*, BS should be stabilized, which commonly includes processes that: (i) decrease the volatile solids and moisture content, presence of pathogen microorganisms, colour and odour^46,47^ and (ii) immobilise/inactivate inorganic contaminants, notably toxic metal(oid)s^48^ and metallic nanoparticles.^34^

Stabilisation of sewage sludge occurs by different physical, chemical and biological treatments, considering economic and regulatory requirements (Fig. 2). In chemical conditioning, alkalization of sewage sludge to inactivate pathogens is a prevalent method, primarily achieved using relatively homogeneous lime-based matrices (CaO; Fig. 2) due to their heightened reactivity and substantial specific heat capacity, which facilitate efficient pathogen eradication.^34^ However, other transformative products of BBA such as relatively more complex BA, owing to their highly alkalinity and reactivity (more in Section 4), have also been validated as effective matrices in sewage sludge management. For instance, studies confirm that the pHpzc (determines the surface charge of the sorbent at a certain pH reaction) of ten different BA are very high, from 9.5 in wood ash to 12.73 in mustard ash.^49,50^ Alkaline disinfection implies the inactivation of pathogens at pH > 12 (ref. 51) which is very common pH reaction of many BA. For instance, addition of fly BA at 1% w/v rate to pH neutral watercourse is able to increase pH reaction by >5.0 pH units in several minutes^49^ reported that incorporating 1% w/v of three distinct fly BA into pH neutral sewage elevated pH reaction to 10.1–12.7, inactivating faecal coliforms and intestinal enterococci while facilitating decolorization. According to Wójcik *et al.*,^52^ the application of wood BA at the high rate of 30 g dm^−3^ dewatered the sewage sludge nearly by 30% and reduced the total bacterial number by >50% *vs.* the raw sewage sludge. Likewise, studies by Lim *et al.*^53^ and Wójcik *et al.*^54^ have shown enhanced sewage sludge dewatering and microbial quality through pathogen reduction following the ash addition.^55,56^

However, if the pH of disinfected sewage sludge falls below 9.5 prior application, there is an additional consideration for dealing with pathogen contamination. Empirical studies have shown that mixing ash with sewage sludge can also improve soil fertility and promote the growth of various plants.^57–59^ In addition,^60^ have shown that the incorporation of fly ash into the alkaline stabilisation process of sewage sludge effectively prevents a decrease in pH and the subsequent re-emergence of pathogens over a period of two months. In a field trial,^61^ investigated the effects of ash-sludge mixtures on wheat and observed a significant increase in grain yield and biomass production. In particular, the nitrogen uptake of wheat was significantly increased by the ash-sludge mixtures, especially at higher sludge application rates. More recently,^58^ investigated the influence of these mixtures on the yield and quality of grass legumes in a six-year field experiment. It was recorded that the ash-sewage sludge mixture can substantially enhance soil fertility, increase plant biomass and uptake of potassium and magnesium. However, even stabilised and aged BS are often (over)loaded with a range of persistent toxic (i) inorganic (metals/metalloids, metallic nanoparticles)^62^ and (ii) emergent organic (furans, halogens, nonylphenols and nonylphenol ethoxylates, polyaromatic hydrocarbons, linear alkylbenzenesulfonates polychlorinated biphenyls, polychlorinated dibenzo-*p*-dioxins) contaminants,^44^ hardly to be immobilised or removed from the BS matrix.

**Fig. 3:**
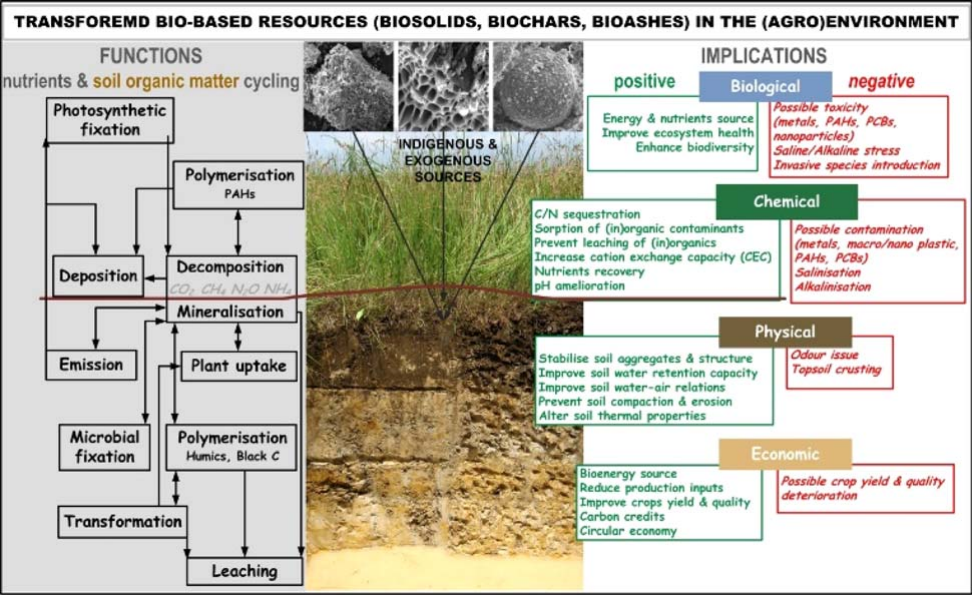
Some of the most important transformative solutions of bio-based resources (BBR) and their environmental functions and implications. Reproduced (adapted) with permission from ref. 5. Copyright© 2021, Elsevier, ref. 64 Copyright© 2019, Elsevier, and ref. 65 Copyright© 2012, Springer.

Presence of such contaminants in the BS has restricted their use in agroecosystems,^63^ continuously improving their use and management. In addition, substantial knowledge gaps persist in comprehending the transport and environmental implications of emerging contaminants and their metabolites, including metallic nanoparticles or organic per- and polyfluoroalkyl substances (PFAS), which remain unaddressed by existing regulations.^34^ Therefore, beside of some traditional solutions in BS management (landfilling, land application, composting), treatments such as pyrolysis (Section 3) or incineration (Section 4) have been recognized as viable options in BS management. These approaches offer efficient ways to reduce the volume of BS, eliminate pathogens and reduce vector attraction, generating additional value-added products (Fig. 3).

## 3. Biochar (BC): an intermediate level in transformation of bio-based resource (BBR)

**Fig. 4:**
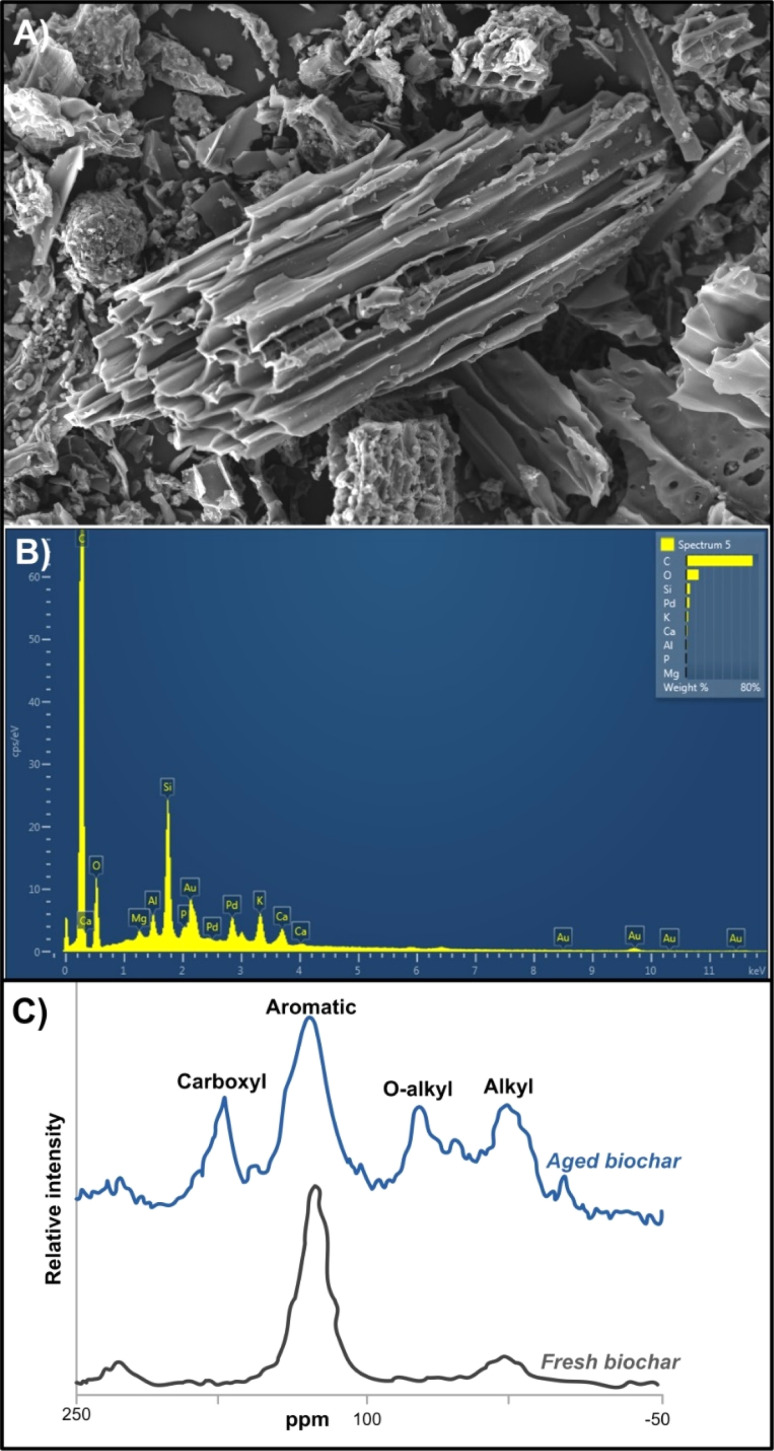
Hardwood-derived biochar (BC) characterised by scanning electron microscopy – SEM (A) and energy dispersive X-ray spectroscopy – EDX (B), with a solid-state ^13^C CP-MAS NMR spectra of fresh and aged BC (C). Reproduced (adapted) with permission from ref. 78 Copyright© 2015, Taylor & Francis, and Ondrasek *et al.*, unpublished Kliknite ili dodirnite ovdje da biste unijeli tekst.

Thermochemical transformation of BBR (*e.g.* agricultural or forest waste/residues) can be performed through different stages and processes such as rapid or pyrolysis, torrefaction, hydrothermal or flash carbonization and gasification.^66^ Pyrolysis occurs in the absence of O_2_ and at relatively lower temperatures (*vs.* incineration), resulting in the formation of a very complex biochar (BC) matrix^67^ (Fig. 1). Both conversions, either pyrolysis or incineration (Section 4) do not only reduce the volume of BBR but also generate additional products and energy (Fig. 1).^68^ The primary applications of BC are intertwined with its numerous beneficial impacts on chemical, physical and biological pedovariables, encompassing enhancements in (i) water and air relations,^69^ (ii) nutrient and pH recovery,^70^ (iii) cation exchange capacity (CEC),^71^ (iv) rhizosphere microbial biodiversity and activity,^72^ (v) crops biomass and yield performances,^73^ (vi) soil remediation,^74^ (vii) catalysis, and materials for energy storage and generation.^7^ Biosolid-derived BC may exhibit distinct properties compared to those derived from biomass, potentially leading to variations in their applications. For instance, BS-derived BC serves as a P source, holding promise for replacing soluble mineral fertilizers, as it can enhance P availability by 38-fold,^75^ thereby fostering increased crop yields.^76^ Generally, alkaline pH reaction of BC may be attributed to elevated concentrations of alkali constitutes (Ca, Mg, K oxides^77^) Nevertheless, the impact of biochar on soil pH depend on various parameters, including the applied dose and pH of BC, initial soil pH reaction, and the buffering capacity (CEC) of soil and BC matrix.^7^ The application of BC has been shown to increase the soil CEC, owing to its large specific surface area and abundance of functional groups (Fig. 4). Moreover, recent advancements in nanotechnology have successfully facilitated the creation of various nano-sized forms of synthesized materials^5^ enhancing surface potential, porosity, dispersivity, surface area, and surface functional groups in many modern materials, including nano BC.^72^ Nano BC consists of ultrafine nano-scale particles, a negatively charged surface, reactive organic species, free radicals, and a surface area surpassing its size^79^ (Fig. 4). Such nano-based performances improve BC mobility in various media and its stability in colloidal form. Notably, NB's large surface area relative to its size and negative surface charge make it an effective adsorbent for nutrients and immobilizing agents of chemicals or hazardous substances in diverse environments, particularly contaminated soil and water matrices^80^ (Fig. 4). For instance, magnetized nano BC (coated nano BC with iron oxide particles) enhances both magnetism induction and availability of active sites for contaminants removal, thereby facilitating easy separation from solution and boosting adsorption capacity.^66^

Nutrient adsorption capability was demonstrated by ref. 81, who observed a reduction in nitrate losses (up to 60%) after application of nano BC. Similarly,^82^ found that application of nano BC decreased N surface translocation to subsurface layers, elevated K content in the topsoil, and consequently increased maize grain and total yield. However, recent findings by ref. 73 reveal that a relatively lower dose (12.5%) of manure-derived nano BC demonstrated beneficial impacts on soil microbiomes, their activity, nutrient mineralization, and the mineral performance of test corn plants in fertilizer mixtures. Conversely, at relatively higher nano BC rates, adverse effects can be expected, particularly on microbial biomass and nutrient uptake. In addition, the incorporation of nano BC into fertilizer mixtures showed promise in mitigating NH_3_ emissions.^83^ These findings emphasize the importance of careful consideration of nano BC concentration levels and their implications for soil health and crop productivity in agricultural practices, warranting further research to optimize BC application strategies for sustainable (agro)ecosystems.

Namely, specific physicochemical properties, such as the presence of numerous active radicals/groups, including aliphatic (C–H) and aromatic carbon (C=C), hydroxyl (–OH), carboxyl (C=O), sulfonyl (S=O), ester (C–O–C), N–H (aliphatic amines), and C≡N–R (nitrile) have confirmed BC's promising role as matrices in environmental protection^73^ (Fig. 4). It was confirmed that soil amelioration with BC can shift the rhizosphere biogeochemistry (*e.g.* from acidic to neutral or alkaline), providing suitable conditions for immobilization of toxic metallic forms and subsequently reducing heavy metal bioavailability and transfer to biota (Fig. 4). Additional mechanisms of contaminates immobilisations are related to electrostatic interactions, ionic exchange and the specific binding of by surface ligands^84^ (Fig. 4). For instance,^84^ observed a reduction in Cd phytotoxicity after application of nano BC to *Brassica chinensis* L. grown in Cd-contaminated pedosphere. In addition, the same authors noted that BC significantly enhanced microbial biomass, abundance and diversity of beneficial microbes for reclamation of metal-contaminated soils (*Actinobacteria*, *Bacteroidetes*), while concurrently reducing the diversity of *Proteobacteria*, which exhibited greater persistence in metal-contaminated soil compared to the control without BC addition.

## 4. Bioash (BA): a final level in transformation of bio-based resources (BBR)

The transition from fossil fuels to green energy, along with the promotion of renewable and sustainable energy sources, has led to a growing reliance on agro-/forest-originated biomass resources.^85^ For instance, in Croatia potentially available agro-biomass is estimated at ∼3.1 Mt per year, with the energy potential of 14 206 GW h per year, substituting nearly 90% of the average energy production from the forest biomass and reducing the energy import by almost 20%.^5^ The importance of the potential for energy production from bio renewables is illustrated by China's production of approximately 1.2 Mt of forest-/agri-residues of which a half remains unexploited.^86^ The portion of biorenewables plant facilities is continuously increasing due to growing awareness of environmental sustainability, advancements in biotechnology, and the pursuit of renewable energy sources as alternatives to fossil fuels.^87,88^ Around 60% of agricultural biomass in China is available as a feedstock for biofuel production and as a raw material for the paper industry, while the rest (around 25%) is used as animal feed or for nutrients (around 15%) recovery.^89^ Currently, renewable energy sources account for around 18% of the total global energy supply,^90^ with forest biomass accounting for a significant proportion of this, *e.g.* 70% of the feedstock in the EU.^91^ However, a significant concern stemming from the utilization of BBR in energy production is the simultaneous generation of the by-product bioash (BA), which levels have multiplied within the EU (from 5.6 to 15.5 Mt per year) and globally (nearly 500 Mt per year) during the 2005–2020 period.^88^ BA is concentrated in minerals and nutrients, and has an ultra-alkaline (pH > 12) and low carbon matrix,^5^ although with possible hazardous components such as PAHs, PCBs and/or toxic metal(oid)s.^92^ Despite its diverse structural properties, BA can be effectively utilized to recovery pH reaction of acidic and nutrient-deficient soils (more in Section 6), to remediate contaminated soils in agricultural and forestry ecosystems,^5^ in industry,^93^ as well as in liquid/solid waste treatment processes^49^ (more in Section 7).

### 4.1 Bioash (BA) & its potential in amelioration of soil acidity

**Fig. 5:**
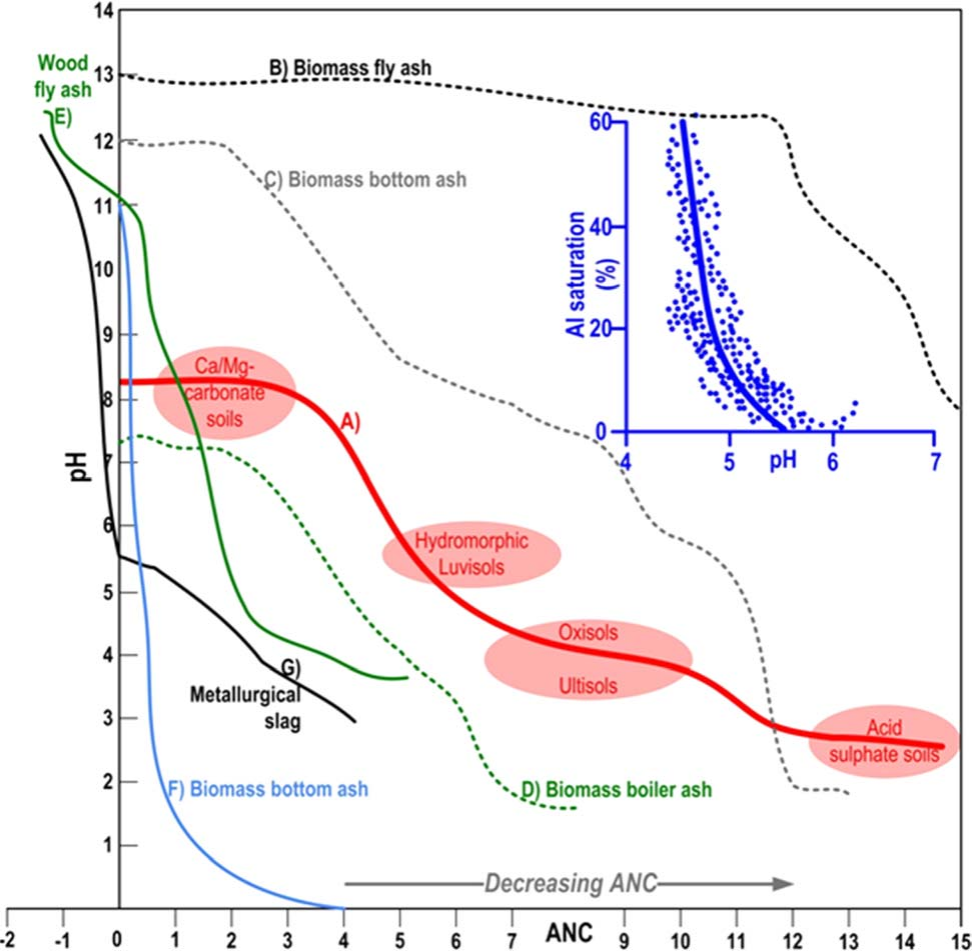
Hypothetical acid neutralising capacity – ANC curves (titration expressed in mL HNO_3_) from well-drained calcareous mineral clayey soils to acid sulfate soils (A).^105,106^ ANC curves of biomass-derived BA (75% wood chips + agro-industrial bio residues) generated from a reciprocating grate combustion (B–D).^107^ ANC curve of forest wood-derived fly BA generated by grate combustion (E).^97^ ANC curve of biomass bottom BA (mixture of crop husks and woody by-products) generated by circulating fluidized-bed combustion (F).^102^ ANC for metallurgical slag (G).^108^ Inserted blue graph represents the soil Al saturation relative to soil CEC at various pH values.^105^

The statistical assessment of physicochemical composition of 37 types (bottom, fly and mixed) of hardwood-derived BA revealed their strongly alkaline pH (11.8–13.1) reaction as a consequence of high content of alkaline (CaO, K_2_O MgO Al_2_O_3_) components.^5^ In the same study (i) bottom BA exhibited elevated concentrations of pozzolanic oxides (SiO_2_, Al_2_O_3_, TiO_2_, Fe_2_O_3_), rendering them suitable for industrial sector (*e.g.* cement production), while (ii) fly BA samples were recognised by higher levels of N and K oxides, and (iii) mixed BA samples contained elevated concentration of P and Ca oxide, as well as Ca carbonate, indicating their capacity for adsorption/precipitation reactions with metals^94^ or certain agrochemicals in alkaline soil/aquatic environments^49^ (more in Section 8). Such observations are in line with well-known wood-originated BA eluates and their pronounced alkaline response (pH 12–13), as a result of dissolution, hydrolysis and weathering of abovementioned alkaline carbonates, bicarbonates, silicates, oxides, hydroxides, silanols, and other basic metal salts.^95–97^ These alkaline constituents are capable of displacing exchangeable H^+^, Mn^2+^, and Al^3+^ from the CEC, thereby influencing soil chemistry.^98,99^ Furthermore, certain ions, notably Al^3+^, can be precipitated and subsequently removed from the soil profile.^100^ Consequently, BA is able to more effectively than some traditional liming conditioners neutralise wide range of acidic pedospheres,^28^ including dystric cambisols,^95^ hydromorphic gleysols,^94^ luvisol,^101^ ultisols,^99^^,^^102^ O-horizon of albic podzols,^103^ calcaric cambisol,^104^ increasing availability of most macro/micronutrients (Fig. 5). Namely, in comparison to other liming materials (*e.g.*, dolomite, limestone, grits and dregs from wood pulp facilities), many studies have shown that BA provides a faster and stronger recovery of the soil pH reaction (by >2 pH units) and a higher acid neutralisation capacity (ANC) than other liming materials.^64,95,109^

**Fig. 6:**
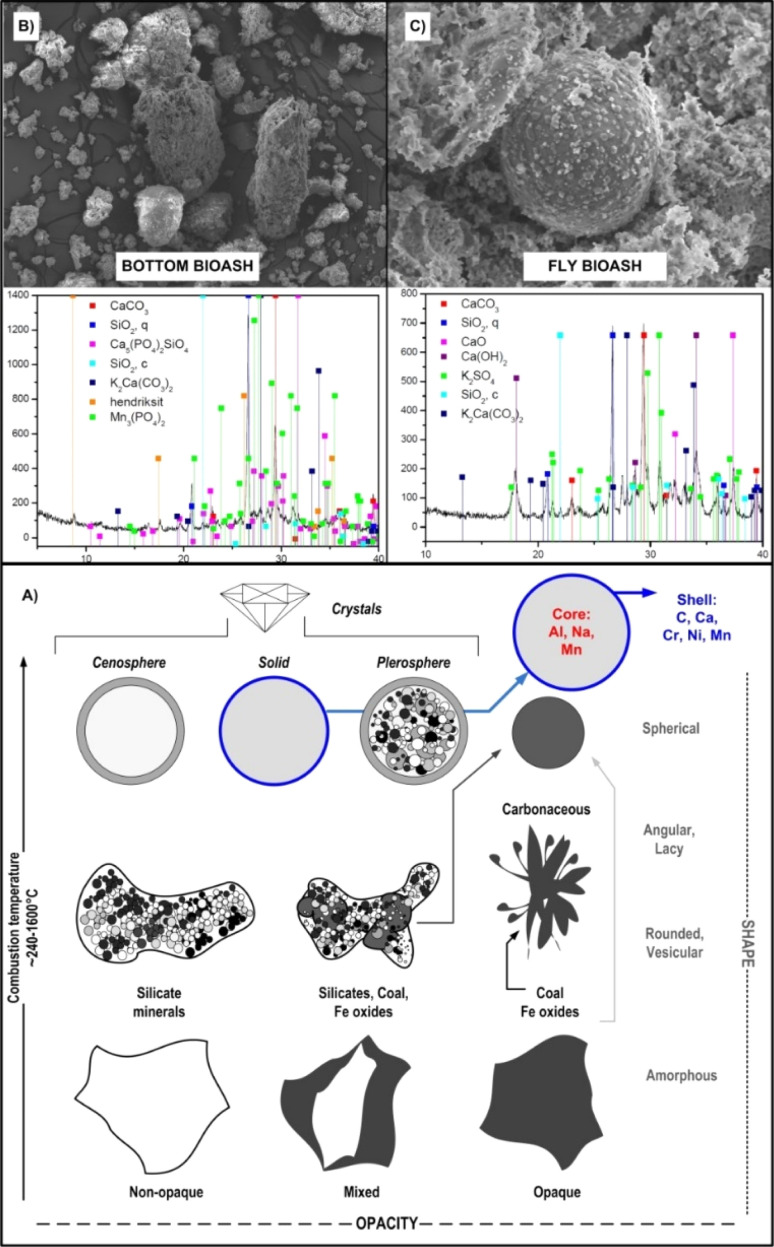
Schematic presentation of the most common morphological classes and specification of bioash (BA) particles (A) with a scanning electron microscopy – SEM and X-ray diffraction – XRD micrographs of bottom BA (B) and fly BA (C). Reproduced (adapted) with permission from ref. 5. Copyright© 2021, Elsevier, ref. 28 Copyright© 2024, Elsevier, ref. 94 Copyright© 2021, Elsevier, and ref. 109 Copyright© 2021, Elsevier.

Due to the relatively lower C content (compared to those of BC and BS) BA are moderately dark to light grey,^110^ diverse in size, morphology and shape, ranging from round, crystalline, angular, amorphous, opaque (solid), magnetic, vesicular, opaque, cenosphere (hollow sphere), pyrophoric (sphere packed with other spheres) to a complex mixture of porous agglomerations with a nanoscale interstice (Fig. 6).

**Fig. 7:**
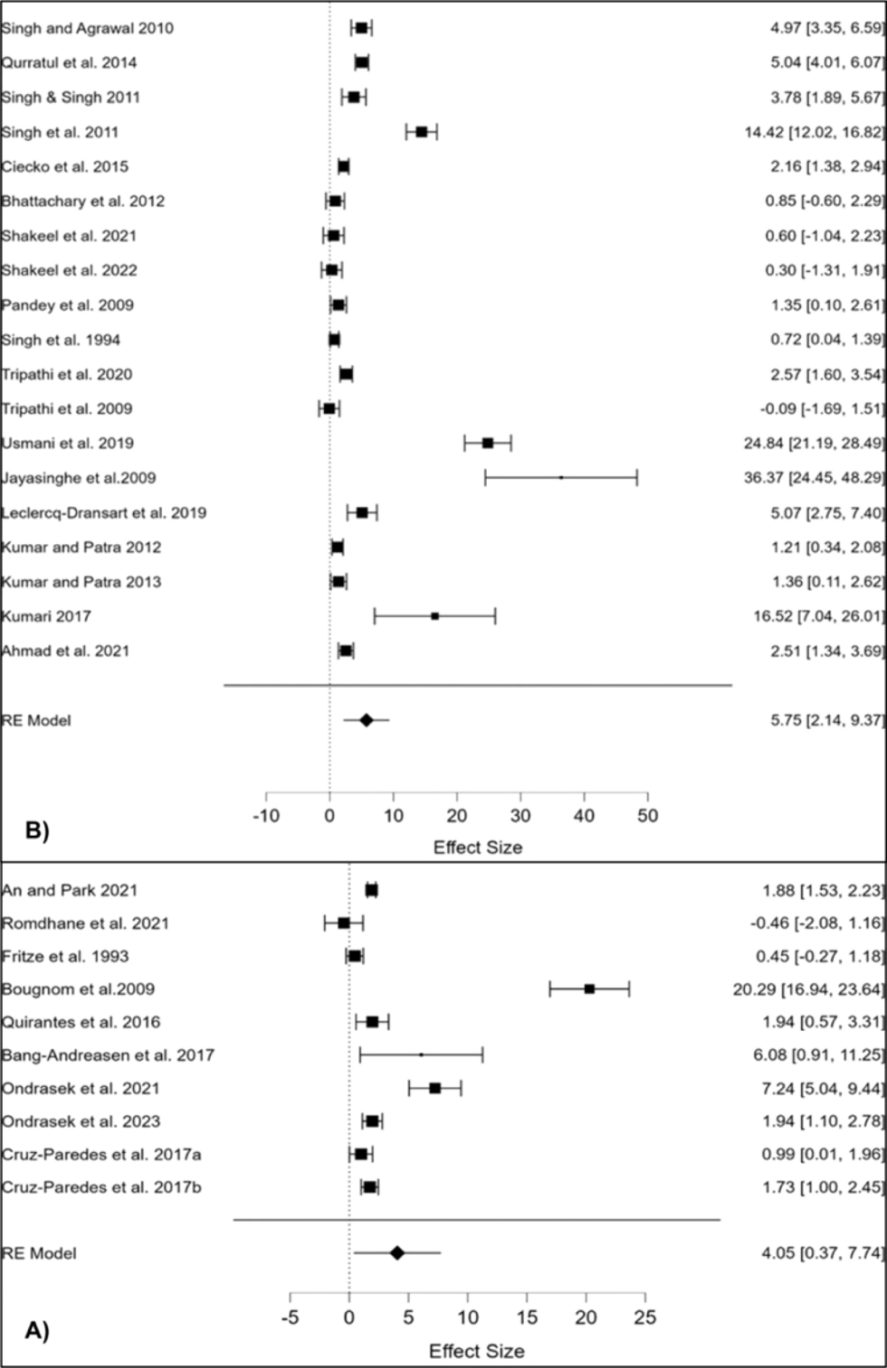
Response of soil pH reaction to fly bioash (A) and coal ash (B) application.^28^

More than 200 types of different minerals and their fractions have been identified in the BA matrix, containing mainly elements such as P, K, Ca, Mg, Si, Zn, Fe, Mn and Al^111^ as revealed by XRD spectra (Fig. 6). For comparison, BA generally contains less pH neutral S-based minerals (*e.g.* arcanite – K_2_SO_4_) than coal-originated ash,^92^ which additionally predispose BA very effective in amelioration of soil acidity (Fig. 7) and/or immobilisation of toxic metals (more in Section 7), given that their dissolution initiates alkaline reactions, neutralizing acidic soils and converting metals into less bioavailable forms.^112^ Furthermore, the quantitative meta-analysis (*n* = 10) revealed a significantly positive overall effect size (Cohen's *d* = 4.05, *p* <0.001, 95% CI: 0.37, 7.74) for fly BA application on soil pH (Fig. 7). In brief, 90% of the effect sizes were positive, confirming a 23% increase in soil pH with fly BA amendment. Additionally, employing the same meta-analytical approach, but with the coal ash matrix (*n* = 19), it was also demonstrated a positive response of soil pH (Cohen's *d* = 5.75, *p* = 0.002, 95% CI: 2.14, 9.37); however, the increase occurred at a slower rate (11%) following coal ash enrichment (Fig. 7).

Generally, chemical composition of BA is conditioned by numerous factors such as: (i) ash type (fly, bottom, cyclonic), (ii) biomass source (hardwood, herbaceous, evergreen, deciduous),^28,113^ (iii) combustion parameters (*e.g.*, temperature, incineration technology^96^) and (iv) post-combustion (wet/dry cooling) processing.^114^ For example, combustion temperatures higher than 400 °C lead to a higher level of carbonisation and promote the aromatic condensation of the degradable aliphatic chains. This is followed by the reduction of oxygen, hydrogen and nitrogen through the processes of dehydration and decarboxylation. These processes lead to an improvement in the structural properties of the material due to greater stability of the carbon bonds.^115^ Thus, it is important to understand the associations among physicochemical BA properties that reveal the fundamentals of biomass properties and improve the predictability of fuel quality (*e.g.*, ref. 89), and allow selecting optimal (post)combustion parameters to eliminate/immobilise pollutants (PCBs, PAHs, toxic metals).

When evaluating the environmental risks of BA its physicochemical properties are crucial for infiltration, salinization, leaching,^116^ deposition, oxidation, and (de)carbonization.^16,117^ By Tao *et al.*^89^ multivariate analytical approach identified a trend of increasing BA and carbon content in the next sequence: woody species > herbaceous dicotyledons > C4 graminoids > C3 graminoids. For instance, BA content in husks (∼18%) can be significantly higher compared to that in wood, which ranges from ∼1.0% in evergreen to ∼1.6% in deciduous trees, or in bark, where it varies from ∼3.0% in evergreens to ∼4.5% in deciduous species. BA from herbaceous and agro-based biomass tends to show less compositional variability and contains comparatively more P, K, S, and Cl. Conversely, wood-derived BA typically has a higher pH, along with elevated concentrations of Mg, Ca, and Mn,^118^ exhibiting higher fluctuations in its composition. Generally, calcium content tends to decrease, while silicon content increases in the order: woody biomass > herbaceous dicotyledons > C4 graminoids > C3 graminoids.^89^

## 5. Bio-based resources (BBR) & implications to biota

Numerous studies confirm that some BBR and/or their derivates contain biologically active substances (Fig. 3) which can induce significant implications to biota, particularly affecting fungi and bacteria^119^ as well as higher plants.^120^ Alterations in bacterial communities and their compositions are mainly due to shifts in soil physicochemical parameters, such as pH elevation, increased electrical conductivity, and enhanced levels of dissolved organic matter and/or nutrient availability^121–125^ (Fig. 3). For instance, a freshly collected fly BA from filters is generally free from pathogens, unlike other potentially hazardous BBR-originated materials such as saturated sludge, sewage sludge/BS or digestate, which are often disposed of in landfills^108,126^ or directly applied without pre/treatment (Fig. 2). Although fresh BA is biologically inert, it can significantly influence a range of biota such as ground vegetation, including mosses and lichens,^127^ native and cultivated forest plants,^120,128^ grasses, herbs, forest fruits and fungi,^128–130^ food crops^131,132^, soil microbiomes^120,133^ mesofauna,^134,135^ and even microbes in wastewater treatment plants.^51^

The application rates and forms of BA are crucial for directing its implications (Fig. 3). For example, large quantities of BA applied to soil can increase the prevalence of fast-growing saprotrophic fungal species from the genera *Mortierella* and *Peziza*, and the order *Hypocreales*.^123,136^ However, when applied BA in rates >5 t ha^−1^ in its non-stabilized form, negative environmental implications are likely.^131^ It is recommended that the maximum application rates of BA should not exceed 10% by weight, as above this threshold, positive environmental implications become unlikely due to: (i) negative impacts on tested species, including reduced germination, growth and yield, diminished carbohydrate content, and symptoms of chlorosis or necrosis^109,137,138^ (ii) potential ionic phytotoxicity,^138,139^ (iii) induced salt/alkali stress, (iv) induced oxidative stress,^140^ (v) possible carcinogenic, mutagenic, and cytotoxic effects due to the presence of PAHs and/or PCBs^131,141–143^ (vi) induced metal(oid)s stress^144^ and (vii) elevated risk of metal transfer to food.^109^

A great obstacle in wider application of some BBR-derived matrices represents the content of PAHs and PCBs (Fig. 3). PAHs are produced through inefficient combustion of hydrocarbons and being recognized as mutagens and carcinogens which can accumulate in plants,^145^ and could be in elevated concentrations in BS,^146^ BC^147^ and BA.^109,135,141,148^ For instance, adding 8 t ha^−1^ of wood-derived BA can increase PAHs concentration in the forest surface Oa soil horizon by up to 6-fold, while retaining PCBs concentration stable in the same layer, but decreasing PCBs level by about 30% in the sub-surface Oi/Oe layer.^143^ This reduction may be attributed to preferential fluxes and the alkaline mobilization of dissolved organic matter, which can act as carriers for PCBs.^143^

Recent studies indicate that while (i) bottom BA can enhance vegetative growth of radish (*Raphanus sativus* L.), it is also able to increase the risk of the cadmium soil-to-plant transfer,^109^ whereas (ii) fly BA can can also improve vegetative growth of maize (*Zea mays* L.) at ≤1.25% w/w rate, but trigger alkaline stress at rates >5% w/w.^49,94^ Moreover, fly ash has been observed to suppress earthworm cocoon production, affect epigeic earthworm populations, and generally impact other soil biota crucial for ecosystem services such as litter processing, soil organic matter decomposition, and nutrient cycling.^134,140^ Nevertheless, even at relatively low rates (*e.g.*, 233 kg ha^−1^), the benefits of BA application persist for longer in metal-contaminated soils, enduring up to 14 years, exhibiting improved soil ecosystem functionality, increased abundance, richness, and diversity of *Diptera*, as well as enhanced microbial enzyme, respiratory, and fungal activity.^135^

## 6. Bio-based resources (BBR) & implications to physicochemical pedovariables

Studies have shown that bottom ash, with its elevated silicon content, can enhance the physical and mechanical properties of heavy clay soils. For instance,^149^ observed that incorporating 5% v/v fly ash into clay soils increased their hydraulic conductivity; however, adding 20% v/v in calcareous soils and 10% v/v in acidic soils led to a significant deterioration in pedovariables. Additionally,^150^ reported that adding 15% w/w fly ash to clay soil significantly decreased the bulk density and improved soil structure, enhancing porosity, workability, root penetration, and water retention. Furthermore, the application of S–Ca and Si–Al fly ash was found to be effective in reducing soil bulk density even after 14 years, according to Leclercq-Dransart *et al.*^140^ In highly expansive and plastic soft soils (*e.g.*, those sensitive to moisture fluctuations and prone to volumetric changes like cracking and shrinkage), the use of various types of ash has resulted in soil stabilization and improved consistency. The plasticity index decreased along with soil dry density, making the soil coarser compared to its original state. These benefits are attributed to the lower density of rice husk-derived BA, reduced compressibility, increased consolidation rate and volume stability, and the pozzolanic reactivity and interactions with soil particles.^151–153^

BBR and their derivates represent excellent precursors for development of combined organo-mineral fertilisers (Fig. 1) which can increase soil productivity. For instance, combining BC with urea significantly enhances production and effectively reducing nitrogen input,^154^ whereas incorporating BC composites with organo-mineral substances can additionally enhance the benefits of fertilizers^59,66^ developed fertilizer aggregates by blending wood and peat-derived BA from with specific proportions of BS and lime. This mixture significantly increased the nitrogen content by >22-fold (from 120 to 2690 mg N kg^−1^). Additionally, by varying the ratios of lime, sewage sludge, and fly ash, the same study succeeded in developing a new co-granulated fertilizer that varied in macro and micro nutrient levels. Furthermore,^155^ demonstrated that the quality of various BAs could be enhanced through co-incineration with BS. This process transformed relatively unavailable phosphorus (AlPO_4_) into more accessible mineral forms such as Ca_2_P_2_O_7_, Ca_5_(PO_4_)_3_Cl, Ca_4_Mg_5_(PO_4_)_6_, and Ca_3_(PO_4_)_2_, which are highly beneficial for use in fertilizers or as soil amendments. Additionally, BC can obtain positive impacts on pedovariables, enhancing water holding capacity, permeability and fertility^66^ ultimately increasing crop yields.^156^ It was shown that BC can enhance the availability macro/micro nutrients (*e.g.*, phosphorus, nitrogen, copper),^73^ while also reducing phosphorus fixation, nitrogen leaching and N_2_O emissions.^73,157^

## 7. Bio-based resources (BBR) & implications to metals remediation

**Fig. 8:**
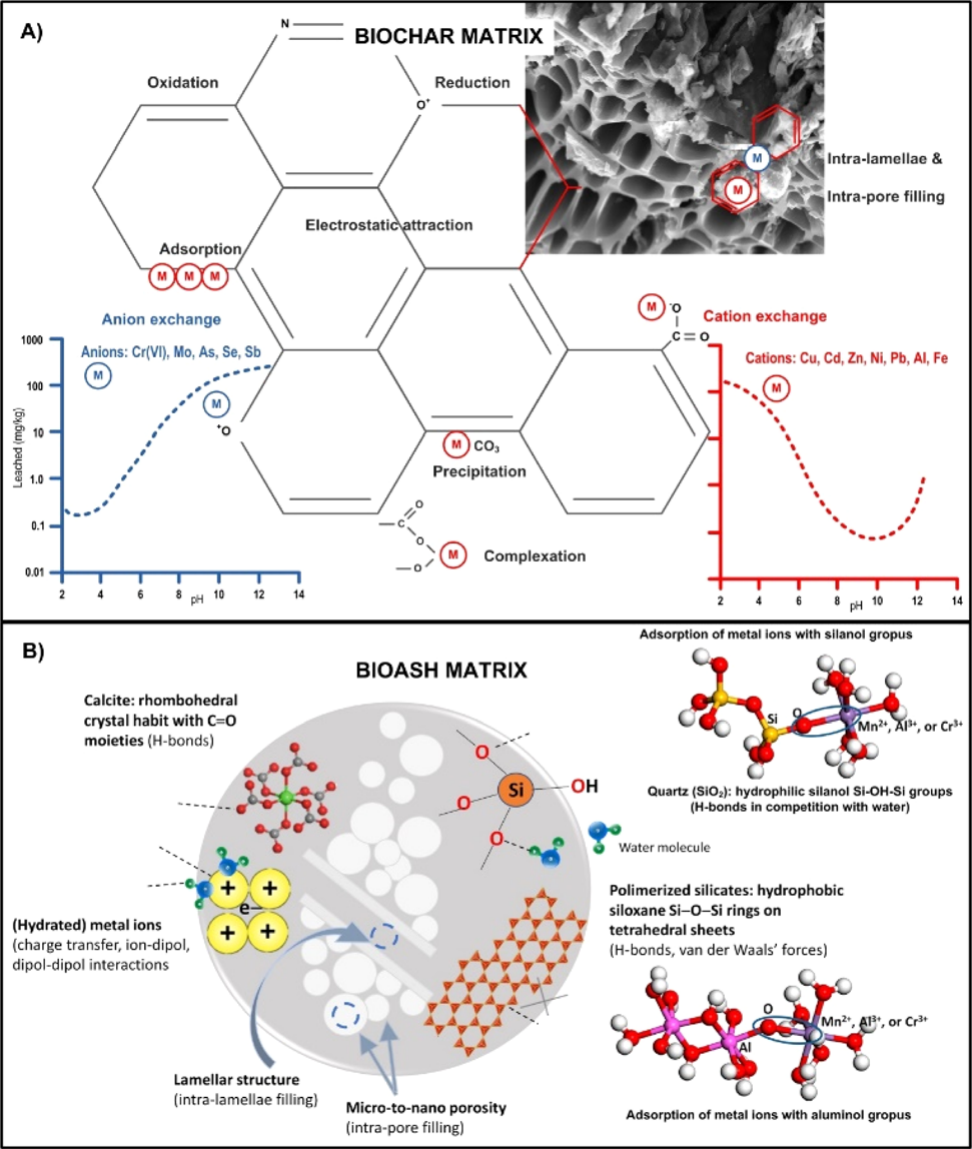
Schematic representation of the most relevant biogeochemical reactions and immobilisation mechanisms of metal(oid)s under varying pH range in the presence of biochar (A) and bioash (B) matrix. Reproduced (adapted) with permission from ref. 28. Copyright© 2024, ref. 49 Copyright© 2021, Springer, and ref. 162 Copyright© 2021, Elsevier.

BBR rich in Si-containing minerals play a key role in the initiation of soil biogeochemistry.^158^ The adsorption function of SiO_2_ and Si/C ratio have been confirmed in many studies as the most important chemical parameter for predicting the adsorption capacity of some BBR,^50,159^ where enrichment with Si implicates a higher adsorption capacity and shorter equilibrium time. It was found that the soil type and the origin of some BBR is a very influential factor for the Si content in BA. For example, the Si content in Indian rice husk BA can be several folds higher than that in Malaysian BA.^159,160^ In addition, the highest SiO_2_ content was found in rice husk ash (82%), followed by wheat ash (59%) and corn ash (41%).^159^ Another important and abundant constituent of BA is crystalline aluminosilicates such as zeolites, build from primary tetrahedral Si(iv)O_4_, Al(iii)O_4_ or P(v)O_4_ units, forming the nanostructured network with excellent intra-porosity and capillarity^161^ that serves as physicochemical sieves (Fig. 8). Such nano-porous structure of zeolites makes them efficient ion-exchangers for ad/desorption and separation of cations and/or (non)polar particles from aqueous media,^163^*i.e.* decreasing bioavailable metallic pools in the soil solution phase (more in the text below) or adsorbing some agrochemicals (more in Section 8).

The study conducted by Dijkstra *et al.*,^164^ confirmed that the concentrations of metals such as Zn, Cu, Ni, Pb and Cd are significantly lower after leaching in contaminated soils compared to the total concentrations and follow pH-sensitive “u”-shaped leaching curves with pronounced differences in the concentrations of the leached metals (Fig. 8). Furthermore, similar modelling studies revealed that the presence of free and highly mobile metal species such as Zn^2+^ and Cd^2+^ was low in alkaline rhizosphere soils, while the concentrations of less bioavailable species (such as carbonate, chloride and organic complexes) or less accessible mineral forms were elevated (*e.g.* malachite for Cu, hydrozincite/smithsonite for Zn, ottavite for Cd).^65^ With regard to the dissolution and adsorption processes of metals in soil and their pH dependence,^87,165^ have shown that the release of cationic metal forms increases with lower pH values, while the release of anions increases with higher pH values or remains independent of pH for some salts (Fig. 8).

The addition of alkaline BA matrices enriched with Ca/K/Mn/Fe/Al/Si (hydro)oxides and/or carbonates to an acidic soil or water environment poor in nutrients and organic matter can significantly affect the mobility of metals and their transfer to biota.^104^ For example, in metal-contaminated soils to which 5% w/w of mixed fly ash (wood and coal) was applied, the leaching of Pb and Cu decreased by more than 87% and 91%, respectively, increasing the number of chemosorption sites and changes in soil pH_H_2_O_ from 4.1 to 6.8.^166^ This also reduced the uptake of both metals by plants and bacteria, which reduced their toxicity.^166^ In addition, studies have shown that the combined use of peat-derived BA and coal fly was more efficient in chemisorption of Cu and Pb than their separate use. Similarly, bottom ash exhibited also high efficiency in removing various metals from aqueous solutions (Fe > Cu > Zn > Mn), likely due to rise in liquid pH (from 4.2 to 8.0), which altered the biogeochemistry of metals toward physical adsorption and/or chemical deposition at the ash interfaces^167^ (Fig. 8).

It was confirmed that remediation and amelioration capacity of BA materials depend on their specific type properties. For instance, adsorption mechanisms between wood-derived bottom BA and fly BA differ significantly, and these can be effectively predicted by Langmuir and pseudo-second-order kinetic model.^168^ Accordingly, fly BA demonstrated a metal adsorption potential by over 4-fold higher than that of bottom BA, what can be explained by higher ability for complexation and precipitation with Si-/OH-based compounds over a broader pH spectrum (2–6). On the other hand, bottom BA exhibit more efficient adsorption in a slightly acidic pH range (5–6), outperforming surface complexation mechanisms (such as those involving aromatic C=C radicals in BC or activated carbon) and crystallisation of carbonate and phosphate anions.^169–172^ Such alterations can impact the mobility of metals and their soil-to-crop transfer. For instance,^109^ applying BA or dolomite to acidic metal-uncontaminated Cambisol recorded enhanced Cd phyto-accumulation (despite no alteration in the total soil Cd concentration in soil), with significant rise of soil pH in both treatments. Additionally,^173^ reported that the addition of 1% w/w fly ash (or dolomite) to Cd/Pb contaminated soils increased mobile and potentially mobile Cd in soil, resulting in almost 50% higher Cd phyto-accumulation. However, these results contradict numerous reports suggesting that the addition of ash or Ca/Mg carbonate matrices can immobilise Cd in uncontaminated soils,^168,174–177^ and with pH-dependent metal biogeochemistry (Fig. 4). Therefore, other mechanisms of metal mobility such as reactivation or CEC supersaturation should be considered before using BA materials in soil chemical ameliorations.

## 8. Bio-based resources (BBR) & implications to pesticides transformation

The adsorption mechanisms explained for metals (Fig. 8) are applicable to many other (in)organic chemicals. Several groups of adsorbents have been investigated extensively so far regarding the removal of pesticides from the (agro)environments: synthetic materials (modified bentonites, zeolites, Si and metal oxides;^178^ activated C (granular, mesoporous C replicas, modified C filter, charcoal;^179^ industrial wastes (C slurry, blast furnace slag, dust and sludge from petrochemical industry;^180^ BA^118,181,182^ and BC types.^115,178,181,183^

The adsorption capacity of BA and BC matrix varies greatly due to significant differences in their surface properties, including porosity and surface functionality, specific surface area and particle size, which affect the overall surface charge.^184,185^ Although synthetic/commercial adsorbents can achieve large specific surface areas of up to 1570 m^2^ g^−1^, the high cost of production limits their wider use.^178^ In contrast, BBR-transformed BA typically has smaller specific surface areas, usually below 50 m^2^ g^−1^;^186^ however, BA is an effective matrix for sorption and can be further optimized to improve the retention of chemicals in soil (Fig. 8). Despite the general assumption that small particles have a larger specific surface area than large particles,^181^ study performed by Deokar *et al.*^187^ confirmed that baggase-derived BA with larger particles can achieve a higher total surface area (51 m^2^ g^−1^), resulting in a higher adsorption capacity for the herbicide 2,4-D, with a removal efficiency of about 90%. However, rice husk-derived BA with a smaller specific surface area (33 m^2^ g^−1^) achieves also high removal of the same herbicide of about 80%. Both BA types have a predominantly mesoporous structure, but with differences in pore geometry. Namely, larger BA particles have been shown to have deeper cylindrical pores with a larger pore volume and internal surface area, allowing them to trap a greater amount of pesticide compared to smaller particles with shallower pores (Fig. 8). In addition, the diffusion of pesticides through inter- and intra-fibrillar (from micro-to nano-sized) capillaries and spaces on the surface of BA and BC, similar to some properties of zeolites, serves as an important adsorption mechanism in porous nanocrystalline aluminosilicate matrices (Fig. 8).

Post-production treatments can significantly enhance the porosity of BA and BC through various methods, including chemical methods involving impregnation with activators, physical methods like crushing or grinding.^115,183^ Functional nanoparticles, such as graphene and its oxides, chitosan, carbon nanotubes, layered double hydroxides, when coated onto BC matrix, produce composite nanomaterials that can effectively remove various pollutants.^66^ Additionally, treating fly BA with NaOH can increase its specific surface area to more than 40 m^2^ g^−1^.^188^ Owing to its meso/microporous structure and high Si–Al content, BA matrix serves as an excellent base for producing micro structured zeolites, recognized as effective adsorbents for ionizing pesticides and other hazardous substances.^189^ Moreover, the adsorption characteristics of BA matrices can be enhanced by microwave irradiation or acid oxidation, which increase pore volume and the specific surface area.^190^

The improvement of the adsorption of ionising and polar pesticides can be achieved by the chemical modification of the BA surface with certain reactive oxygen-containing radicals, and by changing the surface charge and pH of the surrounding media. These may include charge-assisted hydrogen bonding, hydrophobic bonding, ion exchange, charge transfer metal complexation, inner sphere complexation, π-interactions and precipitation.^49^ For instance,^115^ showed that surface oxidation of BC matrix with 3% H_2_O_2_ increased its surface area nearly by 5-fold (from 47 to 140 m^2^ g^−1^) and depleted the surface pH from 7.9 to 4.8, increasing adsorption of the ionising herbicide cyhalofop from 13.9 to 48.3 mg kg^−1^. However, the surface activation of BC had no effect on the adsorption of the polar and non-ionising herbicide clomazone.

Numerous studies have shown that the Si–C–C ratio is essential for determining the adsorption capacity of BA for pesticides; a lower ratio typically results in greater adsorption capacity and longer equilibrium times.^182,187^ Adsorbents rich in silica, such as wheat and corn BA or rice husks, typically exhibit a negative surface charge in aqueous solutions at pH values above 2.2, the point of zero charge (pHpzc), enhancing their ability to bind cations. The effectiveness of pesticide adsorption is influenced by factors such as the amount of used BA, ambient temperature, and contact time with the active substance of the pesticide.^159,191^ Additionally, an increase in the ionic strength of the solution and a higher concentration of pesticides can decrease pesticide removal due to competitive interactions at the BA interface.

The adsorption equilibrium time in porous matrices such as BA is mainly related to the surface area and molecular diffusion into the pores, with large pores (compared to small diameter pores) exhibiting faster adsorption kinetics^159^ (Fig. 8). The adsorption kinetics of BA are usually best fitted to a first–order equation and, in the case of chemisorption, to a pseudo-second-order equation.^191,192^ Due to faster adsorption compared to most synthetic and commercial adsorbents, BA matrices can be used for rapid remediation of pesticide-contaminated water.^178^ For example, adsorption of the herbicide 2,4-D by granular activated C reached equilibrium after 96 hours, while for rice husk, wheat straw and bagasse BA, equilibrium was reached in 15, 120 and 240 minutes, respectively.^159,191,192^

Mass balance estimates from adsorption–desorption experiments with triazine, carbamate and anilide classes of herbicides showed that herbicide adsorption increased with increasing fly ash content in the mixture and reached complete adsorption in samples with “pure” (100%) fly ash.^193^ The desorption of acetone herbicides indicates that the retention on fly ash is mainly due to weak physical interactions such as London dispersion forces.^194^ pointed out that the rapid alkaline degradation of the non-ionising insecticide chlorpyrifos could find application in water/wastewater treatment, as its half-life decreases from 150 hours to 27 hours when the pH increases from 7 to 11. Most pesticide molecules are stable in aqueous suspensions within the pH range of 4 to 9, but become unstable under alkaline conditions due to rapid hydrolysis. Recently, it was confirmed that hardwood fly BA strongly impacts the dissipation dynamics and adsorption mechanism of widely used herbicide terbuthylazine. For instance, within 48 hours of adding BA at a concentration of 1% w/v to the watercourse matrix, terbuthylazine was completely eliminated from its initial concentration (250-fold higher than the EU limit for drinking water), whereas in the treatment without BA, approximately 80% of the initial TBA level persisted in the tested watercourse.^49^ In the same study adsorption kinetics were clearly described by the pseudo-second order, assuming single-layer chemisorption as a rate-controlling mechanism, but multilayer physical sorption and intra-pore diffusion should not be disregarded (Fig. 8).

## 9. Conclusions

Among the diverse array of physical and thermochemical transformations (drying, composting, pyrolysis, incineration) of agricultural and forest residues, as well as municipal and industrial waste streams (BBR), biosolids (BS), biochars (BC) and bioashes (BA) have been intensively studied recently due to their intricate organo-mineral matrix, diverse structures, and significant potential for a wide range of applications in (agro)ecosystems. These matrices are able to induce rapid, strong, and long-lasting effects on various (i) chemical (pH regulation, nutrient content, and organic matter levels), (ii) physical (porosity, bulk density, and water–air dynamics) and microbial (diversity, activity) parameters in soil and waters. In addition, they contribute to numerous environmental services, including the remediation of contaminated sites and wastewater treatment that align with circular economy principles and contemporary waste management strategies/regulations, supporting efforts to mitigate climate change. However, the widespread adoption and scaling up of some of BBR derivatives still encounter certain limitations, including technological constraints in processing and application, concerns regarding (in)organic contamination, toxicity issues as well as non-uniform regulations from inter-national to global levels. By multidisciplinary approach it is possibly to fully unlock the potential of some transformative solutions offered by BBR.

## Consent for publication

All authors agreed for publication.

## Data availability

All data will be available upon request.

## Author contributions

GO conceptualisation, project administration and funding. JH and GO material preparation. JH, SS, JM and GO performed material and statistical data analyses and manuscript drafting. All authors interpreted, discussed and approved the final manuscript.

## Conflicts of interest

The authors declare no competing interest.

## Abbreviations

ANC: Acid neutralisation capacity
BA: bioash
BBR: bio-based resources
BC: biochar
BS: biosolids
CEC: Cation exchange capacity
EDX: Energy dispersive X-ray spectroscopy
EU: European Union
GHG: Greenhouse gases
kg per cap.: kg per capita or person
MSW: Municipal solid waste Mt 10^6^ tons
PAHs: Polycyclic aromatic hydrocarbons
PCBs: Polychlorinated biphenyls
PFAS: Polyfluoroalkyl substances
SEM: Scanning electron microscopy
XRD: X-ray diffraction

## References

[cit1] 70/1 – Transforming our world: the 2030 Agenda for Sustainable Development Transforming our world: the 2030 Agenda for Sustainable Development Preamble, United Nations (UN), 2015, https://sustainabledevelopment.un.org/content/documents/21252030AgendaforSustainableDevelopmentweb.pdf

[cit2] Voukkali I., Papamichael I., Loizia P., Zorpas A. A. (2024). Environ. Sci. Pollut. Res..

[cit3] Wilson D. C. , Global Waste Management Outlook, United Nations Environment Programme and International Solid Waste Association, 2015

[cit4] Gautam M. and Agrawal M., Environmental Footprints and Eco-Design of Products and Processes, 2021, pp. 123–160

[cit5] Ondrasek G., Bubalo Kovačić M., Carević I., Štirmer N., Stipičević S., Udiković-Kolić N., Filipović V., Romić D., Rengel Z. (2021). Renew. Sustainable Energy Rev..

[cit6] Rodriguez D. J. , Serrano H. A., Delgado A., Nolasco D. and Saltiel G., From Waste to Resource: Shifting Paradigms for Smarter Wastewater Interventions in Latin America and the Caribbean, World Bank, Washington, DC, 2020, accessed 10 May 2024

[cit7] Patel S., Kundu S., Halder P., Ratnnayake N., Marzbali M. H., Aktar S., Selezneva E., Paz-Ferreiro J., Surapaneni A., de Figueiredo C. C., Sharma A., Megharaj M., Shah K. (2020). Rev. Environ. Sci. Biotechnol..

[cit8] Abdel-Shafy H. I., Mansour M. S. M. (2018). Egpt. J. Petrol..

[cit9] Zuiderveen E. A. R., Kuipers K. J. J., Caldeira C., Hanssen S. V., van der Hulst M. K., de Jonge M. M. J., Vlysidis A., van Zelm R., Sala S., Huijbregts M. A. J. (2023). Nat. Commun..

[cit10] Pender A., Kelleher L., O’Neill E. (2024). Clean. Circ. Bioecon..

[cit11] Directive 2008/98/EC of the European parliament and the of the council of 19 November 2008 on waste and repealing certain Directives (Text with EEA relevance), 2008, available at: https://eur-lex.europa.eu/legal-content/EN/TXT/?uri=celex%3A32008L0098, last access on 6 May 2024

[cit12] Directive 2009/28/EC of 23 April 2009 on the Promotion of the Use of Energy from Renewable Sources and Amending and Subsequently Repealing Directives 2001/77/EC and 2003/30/EC, European Union, 2009, available at: https://eur-lex.europa.eu/eli/dir/2009/28/oj, last access on 6 May 2024

[cit13] The European Green Deal – European Commission, https://commission.europa.eu/strategy-and-policy/priorities-2019-2024/european-green-deal_en, accessed 10 May 2024

[cit14] Aldieri L., Vinci C. P. (2020). J. Clean. Prod..

[cit15] European Commission , The Rise in Biomass Production and Use Points to a Growing Bioeconomy: Is This Resource Limitless?, European Commission, 2023, available at: https://joint-research-centre.ec.europa.eu/jrc-news-and-updates/rise-biomass-production-and-use-points-growing-bioeconomy-resource-limitless-2023-12-11_en, last access on 10 May 2024

[cit16] Dufossé K., Marie-charlotte M., Augiseau V., Henrion T., Djelal H. (2022). Sustainability.

[cit17] Brazão Farinha C., de Brito J., Veiga R. (2019). Resour., Conserv. Recycl..

[cit18] Sharma K. , Sharma M., Samridhi Kaul K., Singh G. and Arya S. K., Reference Module in Earth Systems and Environmental Sciences, 2023, 10.1016/B978-0-323-93940-9.00086-4

[cit19] UNEP , Report 2019 Executive Summary, United Nations Environment Programme, Nairobi, 2019, Eurostat, Municipal Waste Statistics, 2004 and 2022

[cit20] International Water Management Institute (IWMI) , Scaling Best-Fit Irrigation Bundles in Mali: A Pathway for Improved Development Outcomes. Adaptive Innovation Scaling – Pathways from Small-Scale Irrigation to Sustainable Development, (IWMI Water Issue Brief 23), International Water Management Institute (IWMI), Colombo, Sri Lanka, 2023, p. 8. 10.5337/2023.206

[cit21] Caldeira C., De Laurentiis V., Ghose A., Corrado S., Sala S. (2021). Resour. Conserv. Recycl..

[cit22] Pereira P., Barceló D., Panagos P. (2020). Environ. Res..

[cit23] IPCC , Climate Change 2022: Mitigation of Climate Change. Contribution of Working Group III to the Sixth Assessment Report of the Intergovernmental Panel on Climate Change, 2022

[cit24] Hasan M. M., Rasul M. G., Khan M. M. K., Ashwath N., Jahirul M. I. (2021). Renew. Sustainable Energy Rev..

[cit25] Blasenbauer D., Huber F., Lederer J., Quina M. J., Blanc-Biscarat D., Bogush A., Bontempi E., Blondeau J., Chimenos J. M., Dahlbo H., Fagerqvist J., Giro-Paloma J., Hjelmar O., Hyks J., Keaney J., Lupsea-Toader M., O'Caollai C. J., Orupõld K., Pająk T., Simon F. G., Svecova L., Šyc M., Ulvang R., Vaajasaari K., Van Caneghem J., van Zomeren A., Vasarevičius S., Wégner K., Fellner J. (2020). Waste Manage..

[cit26] Mansor M. and Fatehah M. O., Handbook of Solid Waste Management, 2021, pp. 1–24

[cit27] Chevallier T., Loireau M., Courault R., Chapuis-Lardy L., Desjardins T., Gomez C., Grondin A., Guérin F., Orange D., Pélissier R., Serpantié G., Durand M. H., Derioz P., Gildas Laruelle G., Schwoob M. H., Viovy N., Barrière O., Blanchart E., Blanfort V., Brossard M., Demenois J., Fargette M., Heulin T., Mahe G., Manlay R., Podwojewski P., Rumpel C., Sultan B., Chotte J. L. (2020). Sustainability.

[cit28] Horvatinec J., Buczny J., Ondrasek G. (2024). J. Environ. Manage..

[cit29] Council Directive 86/278/EEC of 12 June 1986 on the protection of the environment, and in particular of the soil, when sewage sludge is used in agriculture, https://eur-lex.europa.eu/legal-content/EN/TXT/?uri=celex%3A31986L0278

[cit30] Upadhyay S. K., Singh G., Rani N., Rajput V. D., Seth C. S., Dwivedi P., Minkina T., Wong M. H., Show P. L., Khoo K. S. (2024). Environ. Technol. Innov..

[cit31] Marchuk S., Tait S., Sinha P., Harris P., Antille D. L., McCabe B. K. (2023). Sci. Total Environ..

[cit32] Ondrasek G. , Kranjčec F., Horvatinec J., Atilija B. and Maurović N., Assessment of Sewage Sludge Quantity at the Disposal Site of the Zagreb Wastewater Treatment Plant. Case Study Report, University of Zagreb Faculty of Agriculture, 2023

[cit33] Pozzebon E. A., Seifert L. (2023). Environ. Health.

[cit34] Le Q., Price G. W. (2024). Waste Manage..

[cit35] Fertilisers in the EU Prices, Trade and Use Contents, 2019, http://ec.europa.eu/agriculture/markets-and-prices/market-briefs/index_en.htm

[cit36] Babcock-Jackson L., Konovalova T., Krogman J. P., Bird R., Díaz L. L. (2023). J. Agric. Food Chem..

[cit37] Andersson K. and Stockholm Environment Institute, Sanitation, Wastewater Management and Sustainability: from Waste Disposal to Resource Recovery, 2021

[cit38] Prywer J., Torzewska A., Cichomski M., Michałowski P. P. (2023). Sci. Rep..

[cit39] Valle S. F., Giroto A. S., Dombinov V., Robles-Aguilar A. A., Jablonowski N. D., Ribeiro C. (2022). Sci. Rep..

[cit40] Gowd S. C., Kumar D., Lin R., Rajendran K. (2022). Bioresour. Technol. Rep..

[cit41] Scholz R. W., Ulrich A. E., Eilittä M., Roy A. (2013). Sci. Total Environ..

[cit42] Kranz C. N., McLaughlin R. A., Johnson A., Miller G., Heitman J. L. (2020). J. Environ. Manage..

[cit43] Elgarahy A. M., Eloffy M. G., Priya A. K., Yogeshwaran V., Yang Z., Elwakeel K. Z., Lopez-Maldonado E. A. (2024). J. Clean. Prod..

[cit44] Lamastra L., Suciu N. A., Trevisan M. (2018). Chem. Biol. Technol. Agric..

[cit45] Australian Bureau of Statistics (ABS), 2021, https://www.abs.gov.au/, accessed 08 May 2024

[cit46] Al-Gheethi A. A., Efaq A. N., Bala J. D., Norli I., Abdel-Monem M. O., Ab. Kadir M. O. (2018). Appl. Water Sci..

[cit47] Arthurson V. (2008). Appl. Environ. Microbiol..

[cit48] Zhang X., Wang X. Q., Wang D. F. (2017). Sustainability.

[cit49] Ondrasek G., Kranjčec F., Maltašić G., Stipičević S. (2021). Biomass Convers. Biorefin..

[cit50] Trivedi N. S., Mandavgane S. A., Mehetre S., Kulkarni B. D. (2016). Environ. Sci. Pollut. Res..

[cit51] Ivanković T., Hrenović J., Itskos G., Koukouzas N., Kovačević D., Milenković J. (2014). Arch. Ind. Hyg. Toxicol..

[cit52] Wójcik M., Stachowicz F., Masłoń A. (2020). Waste Biomass Valorization.

[cit53] Lim S., Jeon W., Lee J., Lee K., Kim N. (2002). Water Res..

[cit54] Wójcik M., Stachowicz F., Masłoń A. (2017). Eng. Protect. Environ..

[cit55] Wong J. W. C., Fang M., Jiang R. (2001). Water Environ. Res..

[cit56] Liu C. , Pathogen Inactivation in Biosolids with Lime and Fly Ash Addition, 2000

[cit57] Shaheen S. M., Hooda P. S., Tsadilas C. D. (2014). J. Environ. Manage..

[cit58] Antonkiewicz J., Popławska A., Kołodziej B., Ciarkowska K., Gambuś F., Bryk M., Babula J. (2020). J. Environ. Manage..

[cit59] Pesonen J., Kuokkanen V., Kuokkanen T., Illikainen M. (2016). J. Environ. Chem. Eng..

[cit60] Kocaer F. O., Alkan U., Baskaya H. S. (2003). Waste Manage. Res..

[cit61] Tsadilas C., Samaras V., Evangelou E., Shaheen S. M. (2014). Int. J. Coal Sci. Technol..

[cit62] Fayiga A., Nwoke O. (2017). Open J. Environ. Biol..

[cit63] Smith S. R. and Smith S. R., Final Report for Discussion. The Implications for Human Health and the Environment of Recycling Biosolids on Agricultural Land, 2008

[cit64] Ondrasek G., Bakić Begić H., Zovko M., Filipović L., Meriño-Gergichevich C., Savić R., Rengel Z. (2019). Sci. Total Environ..

[cit65] Ondrasek G. and Rengel Z., The role of soil organic matter in trace element bioavailability and toxicity, in Abiotic Stress Responses in Plants, 2012, pp. 403–423

[cit66] Chausali N., Saxena J., Prasad R. (2021). J. Agric. Food Res..

[cit67] Osman A. I., Farghali M., Ihara I., Elgarahy A. M., Ayyad A., Mehta N., Ng K. H., Abd El-Monaem E. M., Eltaweil A. S., Hosny M., Hamed S. M., Fawzy S., Yap P. S., Rooney D. W. (2023). Environ. Chem. Lett..

[cit68] Al-Rumaihi A., Shahbaz M., Mckay G., Mackey H., Al-Ansari T. (2022). Renew. Sustainable Energy Rev..

[cit69] Ondrasek G., Rathod S., Manohara K. K., Gireesh C., Anantha M. S., Sakhare A. S., Parmar B., Yadav B. K., Bandumula N., Raihan F., Zielińska-Chmielewska A., Meriño-Gergichevich C., Reyes-Díaz M., Khan A., Panfilova O., Fuentealba A. S., Romero S. M., Nabil B., Wan C., Shepherd J., Horvatinec J. (2022). Plants.

[cit70] Ahmed H. P., Schoenau J. J. (2015). Bioenergy Res..

[cit71] Domingues R. R., Sánchez-Monedero M. A., Spokas K. A., Melo L. C. A., Trugilho P. F., Valenciano M. N., Silva C. A. (2020). Agronomy.

[cit72] Rashid M. I., Shah G. A., Iqbal Z., Ramzan M., Rehan M., Ali N., Shahzad K., Summan A., Ismail I. M. I., Ondrasek G. (2023). Plants.

[cit73] Rashid M. I., Shah G. A., Sadiq M., ul Amin N., Ali A. M., Ondrasek G., Shahzad K. (2023). Plants.

[cit74] Guo M., Song W., Tian J. (2020). Front. Environ. Sci..

[cit75] Yue Y., Shao T., Long X., He T., Gao X., Zhou Z., Liu Z., Rengel Z. (2020). Sci. Total Environ..

[cit76] Faria W. M., de Figueiredo C. C., Coser T. R., Vale A. T., Schneider B. G. (2018). Arch. Agron. Soil Sci..

[cit77] de Figueiredo C. C., Coser T. R., Moreira T. N., Leão T. P., do Vale A. T., Paz-Ferreiro J. (2019). Appl. Sci..

[cit78] Laird D. , Rogovska N.. Biochar effects on nutrient leaching (2nd ed.). Routledge. 2015, https://www.taylorfrancis.com/chapters/edit/10.4324/9780203762264-18/biochar-effects-nutrient-leaching-david-laird-natalia-rogovska

[cit79] Ramanayaka S., Vithanage M., Alessi D. S., Liu W. J., Jayasundera A. C. A., Ok Y. S. (2020). Environ. Sci. Nano.

[cit80] Jiang M., He L., Niazi N. K., Wang H., Gustave W., Vithanage M., Geng K., Shang H., Zhang X., Wang Z. (2023). Biochar.

[cit81] Chen X., Zhou B., Wang Q., Tao W., Lin H. (2020). Catena.

[cit82] Yang Y., Zhou B., Hu Z., Lin H. (2020). Appl. Ecol. Environ. Res..

[cit83] Zhang A., Zhang X., Liang Q., Sun M. (2024). PLoS One.

[cit84] Liu W., Li Y., Feng Y., Qiao J., Zhao H., Xie J., Fang Y., Shen S., Liang S. (2020). Sci. Rep..

[cit85] Strielkowski W., Civín L., Tarkhanova E., Tvaronavičienė M., Petrenko Y. (2021). Energies.

[cit86] Cuiping L., Yanyongjie, Chuangzhi W., Haitao H. (2004). Biomass Bioenergy.

[cit87] Tosti L., van Zomeren A., Pels J. R., Dijkstra J. J., Comans R. N. J. (2019). Chemosphere.

[cit88] Voshell S., Mäkelä M., Dahl O. (2018). Renew. Sustainable Energy Rev..

[cit89] Tao G., Geladi P., Lestander T. A., Xiong S., Geladi P., Xiong S. (2012). Renew. Sustainable Energy Rev..

[cit90] IEA , Resources: Renewables 2019-Global Status Report (REN 21), IEA, Paris, 2019, https://www.iea.org/reports/renewables-2019/power

[cit91] Bioenergy Europe , Statistical Report 2019, Bioenergy Europe, Brussels, 2019

[cit92] Singh A. K. , Masto R. E., Hazra B., Esterle J. and Singh P. K., Ash from Coal and Biomass Combustion, 2020, pp. 91–114

[cit93] Zagvozda M., Dimter S., Rukavina T., Grubeša I. N. (2018). Građevinar.

[cit94] Ondrasek G., Zovko M., Kranjčec F., Savić R., Romić D., Rengel Z. (2021). J. Clean. Prod..

[cit95] Cabral F., Ribeiro H. M., Hilário L., Machado L., Vasconcelos E. (2008). Bioresour. Technol..

[cit96] Pöykiö R., Nurmesniemi H., Dahl O., Mäkelä M. (2014). Trans. Nonferrous Met. Soc. China.

[cit97] Freire M., Lopes H., Tarelho L. A. C. (2015). Waste Manage..

[cit98] Maresca A., Hansen M., Ingerslev M., Astrup T. F. (2018). Biomass Bioenergy.

[cit99] Shi R., Li J., Jiang J., Mehmood K., Liu Y., Xu R., Qian W. (2017). J. Environ. Sci..

[cit100] Li J.-Y., Wang N., Xu R.-K., Tiwari D. (2010). Pedosphere.

[cit101] Ondrasek G., Kranjčec F., Horvatinec J., Bubalo Kovačić M., Husnjak S., Čoga L., Babić D., Rašeta D., Volarić N., Fulajtar E., Rashid M. I., Včev A., Petrinec B. (2023). Agronomy.

[cit102] Shi R. Y., Li J. Y., Xu R. K., Qian W. (2016). Soil Tillage Res..

[cit103] Hansen M., Kepfer-Rojas S., Bjerager P. E. R., Holm P. E., Skov S., Ingerslev M. (2018). For. Ecol. Manage..

[cit104] Quirantes M., Romero E., Nogales R. (2016). Commun. Soil Sci. Plant Anal..

[cit105] Chadwick O. A., Chorover J. (2001). Geoderma.

[cit106] Van Breemen N. , Mulder J. and Driscoll C. T., Acidification and alkalinization of soils

[cit107] Asquer C., Cappai G., Carucci A., De Gioannis G., Muntoni A., Piredda M., Spiga D. (2019). Biomass Bioenergy.

[cit108] Król A., Mizerna K., Bożym M. (2020). J. Hazard. Mater..

[cit109] Ondrasek G., Kranjčec F., Filipović L., Filipović V., Bubalo Kovačić M., Badovinac I. J., Peter R., Petravić M., Macan J., Rengel Z. (2021). Sci. Total Environ..

[cit110] Jalal M., Pouladkhan A., Harandi O. F., Jafari D. (2015). Constr. Build. Mater..

[cit111] Maresca A., Hyks J., Astrup T. F. (2017). Waste Manage..

[cit112] Kindtler N. L., Ekelund F., Rønn R., Kjøller R., Hovmand M., Vestergård M., Christensen S., Johansen J. L. (2019). Environ. Pollut..

[cit113] V Vassilev S., Baxter D., Andersen L. K., Vassileva C. G. (2010). Fuel.

[cit114] Asokbunyarat V., van Hullebusch E. D., Lens P. N. L., Annachhatre A. P. (2015). Water Air Soil Pollut..

[cit115] Gámiz B., Hall K., Spokas K. A., Cox L. (2019). Agronomy.

[cit116] You M., Xu M., Hu Y., Xue S., Zhao J. (2024). ACS Omega.

[cit117] Nunes L. J. R., Matias J. C. O. (2020). Sustainability.

[cit118] Vassilev S. V., Baxter D., Andersen L. K., Vassileva C. G. (2013). Fuel.

[cit119] Ryan D., Karpinska A., Forrestal P. J., Ashekuzzaman S. M., Kakouli-Duarte T., Dowling D. N., Germaine K. J. (2022). Front. Sustainable Food Syst..

[cit120] Saarsalmi A., Smolander A., Kukkola M., Moilanen M., Saramäki J. (2012). For. Ecol. Manage..

[cit121] Frostegård Åand Bååth E., Tunlio A. (1993). Soil Biol. Biochem..

[cit122] Högberg M. N., Högberg P., Myrold D. D. (2007). Oecologia.

[cit123] Bang-Andreasen T., Anwar M. Z., Lanzén A., Kjøller R., Rønn R., Ekelund F., Jacobsen C. S. (2020). FEMS Microbiol. Ecol..

[cit124] Noyce G. L., Fulthorpe R., Gorgolewski A., Hazlett P., Tran H., Basiliko N. (2016). Appl. Soil Ecol..

[cit125] Bang-Andreasen T., Nielsen J. T., Voriskova J., Heise J., Rønn R., Kjøller R., Hansen H. C. B., Jacobsen C. S. (2017). Front. Microbiol..

[cit126] Peng W., Pivato A., Lavagnolo M. C., Raga R. (2018). Waste Manage..

[cit127] Jacobson S., Gustafsson L. (2001). Basic Appl. Ecol..

[cit128] Moilanen M., Silfverberg K., Hokkanen T. J. (2002). For. Ecol. Manage..

[cit129] Olsson B. A., Kellner O. (2002). For. Ecol. Manage..

[cit130] Moilanen M., Fritze H., Nieminen M., Piirainen S., Issakainen J., Piispanen J. (2006). For. Ecol. Manage..

[cit131] Aronsson K. A., Ekelund N. G. A. (2004). J. Environ. Qual..

[cit132] Lopareva-Pohu A., Verdin A., Garçon G., Lounès-Hadj Sahraoui A., Pourrut B., Debiane D., Waterlot C., Laruelle F., Bidar G., Douay F., Shirali P. (2011). Environ. Pollut..

[cit133] Nayak B., Liu R. H., Tang J. (2015). Crit. Rev. Food Sci. Nutr..

[cit134] Grumiaux F., Demuynck S., Pernin C., Leprêtre A. (2015). Ecotoxicol. Environ. Saf..

[cit135] Leclercq-Dransart J., Demuynck S., Bidar G., Douay F., Grumiaux F., Louvel B., Pernin C., Leprêtre A. (2019). Appl. Soil Ecology.

[cit136] Bååth E., Frostegård Å., Pennanen T., Fritze H. (1995). Soil Biol. Biochem..

[cit137] Singh N., Singh S. N., Yunus M., Ahmad K. J. (1994). Ecotoxicology.

[cit138] Singh S. N. N., Kulshreshtha K., Ahmad K. J. J. (1997). Ecol. Eng..

[cit139] Lee D.-S., Lim S.-S., Park H.-J., Yang H. I., Park S.-I., Kwak J.-H., Choi W.-J. (2019). Environ. Int..

[cit140] Leclercq-Dransart J., Demuynck S., Bidar G., Douay F., Grumiaux F., Louvel B., Pernin C., Leprêtre A. (2019). Appl. Soil Ecology.

[cit141] El-Mogazi D., Lisk D. J., Weinstein L. H. (1988). Sci. Total Environ..

[cit142] Ramdahl T., Alfheim I., Rustad S., Olsen T. (1982). Chemosphere.

[cit143] Bundt M., Krauss M., Blaser P., Wilcke W. (2001). J. Environ. Qual..

[cit144] Goix S., Mombo S., Schreck E., Pierart A., Lévêque T., Deola F., Dumat C. (2015). Chemosphere.

[cit145] Ma B., He Y., Hai Chen H., Ming Xu J., Rengel Z. (2010). Environ. Pollut..

[cit146] Chen C. F., Ju Y. R., Lim Y. C., Hsieh S. L., Tsai M. L., Sun P. P., Katiyar R., Chen C. W., Di Dong C. (2019). Int. J. Environ. Res. Public Health.

[cit147] Buss W., Hilber I., Graham M. C., Masěk O. (2022). ACS Sustain. Chem. Eng..

[cit148] Sivaram A. K., Logeshwaran P., Subashchandrabose S. R., Lockington R., Naidu R., Megharaj M. (2018). Sci. Rep..

[cit149] Chang A. C., Lund L. J., Page A. L., Warneke J. E. (1977). J. Environ. Qual..

[cit150] Sahu G., Bag A. G., Chatterjee N., Kumar A. (2017). J. Pharmacogn. Phytochem..

[cit151] Mir B. A., Sridharan A. (2013). Geotech. Geol. Eng..

[cit152] Jafer H., Atherton W., Sadique M., Ruddock F., Loffill E. (2018). Appl. Clay Sci..

[cit153] Sarkar G. (2012). Global J. Res. Eng..

[cit154] Qian L., Chen L., Joseph S., Pan G., Li L., Zheng J., Zhang X., Zheng J., Yu X., Wang J. (2014). Carbon Manag..

[cit155] Zhao Y., Ren Q., Na Y. (2019). Fuel Process. Technol..

[cit156] Liu S. B., Tan X. F., Liu Y. G., Gu Y. L., Zeng G. M., Hu X. J., Wang H., Zhou L., Jiang L. H., Bin Zhao B. (2016). RSC Adv..

[cit157] Pan F., Chapman S. J., Li Y., Yao H. (2017). J. Soils Sediments.

[cit158] Gokhale N. A., Trivedi N. S., Mandavgane S. A., Kulkarni B. D. (2020). Int. J. Environ. Sci. Technol..

[cit159] Deokar S. K., Singh D., Modak S., Mandavgane S. A., Kulkarni B. D. Desalin. Water Treat..

[cit160] Garba J., Samsuri W. A., Othman R., Hamdani M. S. A. (2019). Sci. Rep..

[cit161] Ramanathan S., Gopinath S. C. B., Arshad M. K. M., Poopalan P. (2020). J. Clean. Prod..

[cit162] Li Y., Yu H., Liu L., Yu H. (2021). J. Hazard. Mater..

[cit163] Ghasemi Z., Sourinejad I., Kazemian H., Rohani S. (2018). Rev. Aquac..

[cit164] Dijkstra J. J., Meeussen J. C. L., Comans R. N. J. (2004). Environ. Sci. Technol..

[cit165] van der Sloot D. S. and Kosson H. A., Leaching assessment methodologies for disposal and use of bauxite residuestle, International Aluminium Institute, 2010

[cit166] Kumpiene J., Lagerkvist A., Maurice C. (2007). Environ. Pollut..

[cit167] Asokbunyarat V., Lens P. N. L., Annachhatre A. P. (2015). Water, Air, Soil Pollut..

[cit168] Park J.-H., Eom J.-H., Lee S.-L., Hwang S.-W., Kim S.-H., Kang S.-W., Yun J.-J., Cho J.-S., Lee Y.-H., Seo D.-C. (2020). Sci. Total Environ..

[cit169] Xu X., Schierz A., Xu N., Cao X. (2016). J. Colloid Interface Sci..

[cit170] Cui X., Fang S., Yao Y., Li T., Ni Q., Yang X., He Z. (2016). Sci. Total Environ..

[cit171] Xu Z., Xu X., Tsang D. C. W., Cao X. (2018). Environ. Pollut..

[cit172] V Rechberger M., Kloss S., Wang S.-L., Lehmann J., Rennhofer H., Ottner F., Wriessnig K., Daudin G., Lichtenegger H., Soja G., Zehetner F. (2019). Chemosphere.

[cit173] Shaheen S. M., Rinklebe J. (2015). Ecol. Eng..

[cit174] Boca Santa R. A. A., Soares C., Riella H. G. (2016). J. Hazard. Mater..

[cit175] Seyfferth A. L., Amaral D., Limmer M. A., Guilherme L. R. G. (2019). Environ. Int..

[cit176] Wang T., Xue Y., Zhou M., Liang A., Liu J., Mei M., Lao X., Hou H., Li J. (2020). J. Clean. Prod..

[cit177] Wang M., Wu S., Guo J., Zhang X., Yang Y., Chen F., Zhu R. (2019). J. Hazard. Mater..

[cit178] Trivedi N. S., Mandavgane S. A. (2018). Separat. Purif. Rev..

[cit179] Yargicoglu E. N., Sadasivam B. Y., Reddy K. R., Spokas K. (2015). Waste Manage..

[cit180] Gupta V. K., Ali I., Suhas, Saini V. K. (2006). J. Colloid Interface Sci..

[cit181] Trivedi N. S., Mandavgane S. A., Mehetre S., Kulkarni B. D. (2016). Environ. Sci. Pollut. Res..

[cit182] Gokhale N. A., Trivedi N. S., Mandavgane S. A., Kulkarni B. D. (2020). Int. J. Environ. Sci. Technol..

[cit183] Alam M. M., Hossain M. A., Hossain M. D., Johir M. A. H., Hossen J., Rahman M. S., Zhou J. L., Hasan A. T. M. K., Karmakar A. K., Ahmed M. B. (2020). Processes.

[cit184] Hamidi N. H., Ahmed O. H., Omar L., Ch’ng H. Y. (2021). Agronomy.

[cit185] Amalina F., Razak A. S. A., Krishnan S., Zularisam A. W., Nasrullah M. (2022). Clean. Mater..

[cit186] Li L., Yu C., Bai J., Wang Q., Luo Z. (2012). J. Hazard. Mater..

[cit187] Deokar S. K., Mandavgane S. A., Kulkarni B. D. (2016). Curr. Sci..

[cit188] Koukouzas N., Vasilatos C., Itskos G., Mitsis I., Moutsatsou A. (2010). J. Hazard. Mater..

[cit189] Belviso C. (2018). Prog. Energy Combust. Sci..

[cit190] Hamzah M. H., Ahmad Asri M. F., Che Man H., Mohammed A. (2019). Int. J. Environ. Res. Public Health.

[cit191] Trivedi N. S., Kharkar R. A., Mandavgane S. A. (2019). Waste Biomass Valorization.

[cit192] Deokar S. K., Mandavgane S. A. (2015). Adsorpt. Sci. Technol..

[cit193] Konstantinou I. K., Albanis T. A. (2000). J. Agric. Food Chem..

[cit194] Ukpebor J. E., Ikpeni S., Ejiogu N. C., Ukpebor E. E. (2014). Niger. J. Technol..

